# Connectivity as a universal predictor of tau progression in atypical Alzheimer’s disease

**DOI:** 10.1093/brain/awaf279

**Published:** 2025-08-14

**Authors:** Hannah de Bruin, Colin Groot, Henryk Barthel, Gérard N Bischof, Ganna Blazhenets, Ronald Boellaard, Baayla D C Boon, Matthias Brendel, David M Cash, William Coath, Gregory S Day, Bradford C Dickerson, Elena Doering, Alexander Drzezga, Christopher H van Dyck, Thilo van Eimeren, Wiesje M van der Flier, Carolyn A Fredericks, Tim D Fryer, Elsmarieke van de Giessen, Brian A Gordon, Jonathan Graff-Radford, Lea T Grinberg, Oskar Hansson, Diana A Hobbs, Merle C Hoenig, Günter Höglinger, David J Irwin, P Simon Jones, Keith A Josephs, Yuta Katsumi, Renaud La Joie, Edward B Lee, Johannes Levin, Maura Malpetti, Scott M McGinnis, Adam P Mecca, Rosaleena Mohanty, Ilya M Nasrallah, John T O’Brien, Ryan S O’Dell, Carla Palleis, Robert Perneczky, Jeffrey S Phillips, Deepti Putcha, Gil D Rabinovici, Nesrine Rahmouni, Pedro Rosa-Neto, James B Rowe, Michael Rullmann, Osama Sabri, Dorothee Saur, Andreas Schildan, Jonathan M Schott, Matthias L Schroeter, William W Seeley, Stijn Servaes, Irene Sintini, Ruben Smith, Salvatore Spina, Jenna Stevenson, Erik Stomrud, Olof Strandberg, Joseph Therriault, Pontus Tideman, Alexandra Touroutoglou, Anne E Trainer, Denise Visser, Fattin Wekselman, Philip S J Weston, Jennifer L Whitwell, David A Wolk, Keir Yong, Yolande A L Pijnenburg, Nicolai Franzmeier, Rik Ossenkoppele

**Affiliations:** Alzheimer Center Amsterdam, Neurology, Vrije Universiteit Amsterdam, Amsterdam UMC Location VUmc, Amsterdam 1081 HV, The Netherlands; Amsterdam Neuroscience, Neurodegeneration, Amsterdam 1081 HV, The Netherlands; Institute for Stroke and Dementia Research (ISD), University Hospital, Ludwig Maximilian University of Munich, Munich 81377, Germany; Alzheimer Center Amsterdam, Neurology, Vrije Universiteit Amsterdam, Amsterdam UMC Location VUmc, Amsterdam 1081 HV, The Netherlands; Amsterdam Neuroscience, Neurodegeneration, Amsterdam 1081 HV, The Netherlands; Clinical Memory Research Unit, Department of Clinical Sciences Malmö, Faculty of Medicine, Lund University, Lund 221 00, Sweden; Department of Nuclear Medicine, University of Leipzig, Leipzig 04109, Germany; Research Center Jülich, Institute for Neuroscience and Medicine II, Molecular Organization of the Brain, Jülich 52428, Germany; Faculty of Medicine and University Hospital Cologne, Department of Nuclear Medicine, University of Cologne, Cologne 50923, Germany; Department of Neurology, Memory and Aging Center, Weill Institute for Neurosciences, University of California, San Francisco, San Francisco, CA 94158, USA; Radiology & Nuclear Medicine, Vrije Universiteit Amsterdam, Amsterdam UMC Location VUmc, Amsterdam 1081 HV, The Netherlands; Amsterdam Neuroscience, Brain Imaging, Amsterdam 1081 HV, The Netherlands; Alzheimer Center Amsterdam, Neurology, Vrije Universiteit Amsterdam, Amsterdam UMC Location VUmc, Amsterdam 1081 HV, The Netherlands; Neurology/neuropathology,Mayo Clinic, Jacksonville, FL 32224, USA; Department of Nuclear Medicine, LMU University Hospital, Munich 81377, Germany; Munich Cluster for Systems Neurology (SyNergy), Munich 81377, Germany; German Center for Neurodegenerative Diseases (DZNE), Munich 81377, Germany; German Cancer Consortium (DKTK), Partner Site Munich, a Partnership Between German Cancer Research Center (DKFZ) and LMU Munich, Munich 81377, Germany; Bavarian Cancer Research Center (BZKF), Partner Site Munich, Munich 81377, Germany; Dementia Research Centre, UCL Queen Square Institute of Neurology, London WC1N 3BG, UK; UK Dementia Research Institute, UCL, London NW1 3BT, UK; Dementia Research Centre, UCL Queen Square Institute of Neurology, London WC1N 3BG, UK; Neurology/neuropathology,Mayo Clinic, Jacksonville, FL 32224, USA; Frontotemporal Disorders Unit, Department of Neurology, Massachusetts General Hospital and Harvard Medical School, Boston, MA 02114, USA; Faculty of Medicine and University Hospital Cologne, Department of Nuclear Medicine, University of Cologne, Cologne 50923, Germany; German Center for Neurodegenerative Diseases (DZNE), Bonn/Cologne 53127, Germany; Research Center Jülich, Institute for Neuroscience and Medicine II, Molecular Organization of the Brain, Jülich 52428, Germany; Faculty of Medicine and University Hospital Cologne, Department of Nuclear Medicine, University of Cologne, Cologne 50923, Germany; German Center for Neurodegenerative Diseases (DZNE), Bonn/Cologne 53127, Germany; Alzheimer’s Disease Research Unit, Yale University School of Medicine, New Haven, CT 06510, USA; Department of Psychiatry, Yale University School of Medicine, New Haven, CT 06510, USA; Faculty of Medicine and University Hospital Cologne, Department of Nuclear Medicine, University of Cologne, Cologne 50923, Germany; Department of Neurology, Faculty of Medicine and University Hospital Cologne, University of Cologne, Cologne 50937, Germany; Alzheimer Center Amsterdam, Neurology, Vrije Universiteit Amsterdam, Amsterdam UMC Location VUmc, Amsterdam 1081 HV, The Netherlands; Amsterdam Neuroscience, Neurodegeneration, Amsterdam 1081 HV, The Netherlands; Department of Neurology, Yale University School of Medicine, New Haven, CT 06510, USA; Department of Neurology, Yale New Haven Hospital, New Haven, CT 06510, USA; Department of Clinical Neurosciences and Cambridge University Hospitals NHS Trust, University of Cambridge, Cambridge CB2 0QQ, UK; Wolfson Brain Imaging Centre, University of Cambridge, Cambridge CB2 0QQ, UK; Radiology & Nuclear Medicine, Vrije Universiteit Amsterdam, Amsterdam UMC Location VUmc, Amsterdam 1081 HV, The Netherlands; Amsterdam Neuroscience, Brain Imaging, Amsterdam 1081 HV, The Netherlands; Department of Radiology, Washington University in St Louis, St Louis, MO 63130, USA; Knight Alzheimer’s Disease Research Center, Washington University in St Louis, St Louis, MO 63130, USA; Department of Neurology, Mayo Clinic, Rochester, MN 55905, USA; Department of Neurology, Memory and Aging Center, Weill Institute for Neurosciences, University of California, San Francisco, San Francisco, CA 94158, USA; Department of Pathology, University of California, San Francisco, San Francisco, CA 94143, USA; Clinical Memory Research Unit, Department of Clinical Sciences Malmö, Faculty of Medicine, Lund University, Lund 221 00, Sweden; Department of Radiology, Washington University in St Louis, St Louis, MO 63130, USA; Knight Alzheimer’s Disease Research Center, Washington University in St Louis, St Louis, MO 63130, USA; Research Center Jülich, Institute for Neuroscience and Medicine II, Molecular Organization of the Brain, Jülich 52428, Germany; Faculty of Medicine and University Hospital Cologne, Department of Nuclear Medicine, University of Cologne, Cologne 50923, Germany; Munich Cluster for Systems Neurology (SyNergy), Munich 81377, Germany; German Center for Neurodegenerative Diseases (DZNE), Munich 81377, Germany; Department of Neurology, LMU University Hospital, LMU Munich, Munich 81377, Germany; Department of Neurology, University of Pennsylvania, Philadelphia, PA 19104, USA; Penn Frontotemporal Degeneration Center, University of Pennsylvania, Philadelphia, PA 19104, USA; Department of Clinical Neurosciences and Cambridge University Hospitals NHS Trust, University of Cambridge, Cambridge CB2 0QQ, UK; Department of Neurology, Mayo Clinic, Rochester, MN 55905, USA; Frontotemporal Disorders Unit, Department of Neurology, Massachusetts General Hospital and Harvard Medical School, Boston, MA 02114, USA; Department of Neurology, Memory and Aging Center, Weill Institute for Neurosciences, University of California, San Francisco, San Francisco, CA 94158, USA; Institute on Aging, University of Pennsylvania, Philadelphia, PA 19104, USA; Department of Pathology & Laboratory Medicine, University of Pennsylvania, Philadelphia, PA 19104, USA; Center for Neurodegenerative Disease Research, Perelman School of Medicine, University of Pennsylvania, Philadelphia, PA 19104, USA; Munich Cluster for Systems Neurology (SyNergy), Munich 81377, Germany; German Center for Neurodegenerative Diseases (DZNE), Munich 81377, Germany; Department of Neurology, LMU University Hospital, LMU Munich, Munich 81377, Germany; Department of Clinical Neurosciences and Cambridge University Hospitals NHS Trust, University of Cambridge, Cambridge CB2 0QQ, UK; Department of Clinical Neurosciences, UK Dementia Research Institute at the University of Cambridge, Cambridge CB2 0AH, UK; Frontotemporal Disorders Unit, Department of Neurology, Massachusetts General Hospital and Harvard Medical School, Boston, MA 02114, USA; Alzheimer’s Disease Research Unit, Yale University School of Medicine, New Haven, CT 06510, USA; Department of Psychiatry, Yale University School of Medicine, New Haven, CT 06510, USA; Division of Clinical Geriatrics, Center for Alzheimer Research, Department of Neurobiology, Karolinska Institutet, Huddinge 141 83, Sweden; Department of Radiology, University of Pennsylvania, Philadelphia, PA 19104, USA; Department of Psychiatry, University of Cambridge, Cambridge CB2 0SZ, UK; Alzheimer’s Disease Research Unit, Yale University School of Medicine, New Haven, CT 06510, USA; Department of Psychiatry, Yale University School of Medicine, New Haven, CT 06510, USA; Department of Neurology, LMU University Hospital, LMU Munich, Munich 81377, Germany; Department of Psychiatry and Psychotherapy, LMU University Hospital, Munich 80336, Germany; Department of Neurology, University of Pennsylvania, Philadelphia, PA 19104, USA; Penn Frontotemporal Degeneration Center, University of Pennsylvania, Philadelphia, PA 19104, USA; Frontotemporal Disorders Unit, Department of Neurology, Massachusetts General Hospital and Harvard Medical School, Boston, MA 02114, USA; Department of Neurology, Memory and Aging Center, Weill Institute for Neurosciences, University of California, San Francisco, San Francisco, CA 94158, USA; Department of Radiology and Biomedical Imaging, University of California, San Francisco, San Francisco, CA 94143, USA; McGill Centre for Studies in Aging, Department of Neurology and Neurosurgery, McGill University, Montreal, QC H3A 2B4, Canada; McGill Centre for Studies in Aging, Department of Neurology and Neurosurgery, McGill University, Montreal, QC H3A 2B4, Canada; Department of Clinical Neurosciences and Cambridge University Hospitals NHS Trust, University of Cambridge, Cambridge CB2 0QQ, UK; Medical Research Council Cognition and Brain Sciences Unit, Cambridge CB2 7EF, UK; Department of Nuclear Medicine, University of Leipzig, Leipzig 04109, Germany; Department of Nuclear Medicine, University of Leipzig, Leipzig 04109, Germany; Department of Neurology, University of Leipzig, Leipzig 04103, Germany; Department of Nuclear Medicine, University of Leipzig, Leipzig 04109, Germany; Dementia Research Centre, UCL Queen Square Institute of Neurology, London WC1N 3BG, UK; Clinic for Cognitive Neurology, University of Leipzig, Leipzig 04103, Germany; Department of Neurology, Max Planck Institute for Human Cognitive and Brain Sciences, Leipzig 04103, Germany; Department of Neurology, Memory and Aging Center, Weill Institute for Neurosciences, University of California, San Francisco, San Francisco, CA 94158, USA; Department of Pathology, University of California, San Francisco, San Francisco, CA 94143, USA; McGill Centre for Studies in Aging, Department of Neurology and Neurosurgery, McGill University, Montreal, QC H3A 2B4, Canada; Department of Radiology, Mayo Clinic, Rochester, MN 55905, USA; Clinical Memory Research Unit, Department of Clinical Sciences Malmö, Faculty of Medicine, Lund University, Lund 221 00, Sweden; Memory Clinic, Skåne University Hospital, Malmö 205 02, Sweden; Department of Neurology, Memory and Aging Center, Weill Institute for Neurosciences, University of California, San Francisco, San Francisco, CA 94158, USA; McGill Centre for Studies in Aging, Department of Neurology and Neurosurgery, McGill University, Montreal, QC H3A 2B4, Canada; Clinical Memory Research Unit, Department of Clinical Sciences Malmö, Faculty of Medicine, Lund University, Lund 221 00, Sweden; Memory Clinic, Skåne University Hospital, Malmö 205 02, Sweden; Clinical Memory Research Unit, Department of Clinical Sciences Malmö, Faculty of Medicine, Lund University, Lund 221 00, Sweden; McGill Centre for Studies in Aging, Department of Neurology and Neurosurgery, McGill University, Montreal, QC H3A 2B4, Canada; Clinical Memory Research Unit, Department of Clinical Sciences Malmö, Faculty of Medicine, Lund University, Lund 221 00, Sweden; Memory Clinic, Skåne University Hospital, Malmö 205 02, Sweden; Frontotemporal Disorders Unit, Department of Neurology, Massachusetts General Hospital and Harvard Medical School, Boston, MA 02114, USA; Clinical Neurosciences Imaging Center, Yale University School of Medicine, New Haven, CT 06510, USA; Amsterdam Neuroscience, Neurodegeneration, Amsterdam 1081 HV, The Netherlands; Radiology & Nuclear Medicine, Vrije Universiteit Amsterdam, Amsterdam UMC Location VUmc, Amsterdam 1081 HV, The Netherlands; Amsterdam Neuroscience, Brain Imaging, Amsterdam 1081 HV, The Netherlands; Department of Neurology, Memory and Aging Center, Weill Institute for Neurosciences, University of California, San Francisco, San Francisco, CA 94158, USA; Department of Pathology, University of California, San Francisco, San Francisco, CA 94143, USA; Dementia Research Centre, UCL Queen Square Institute of Neurology, London WC1N 3BG, UK; UK Dementia Research Institute, UCL, London NW1 3BT, UK; Department of Radiology, Mayo Clinic, Rochester, MN 55905, USA; Department of Neurology, University of Pennsylvania, Philadelphia, PA 19104, USA; Institute on Aging, University of Pennsylvania, Philadelphia, PA 19104, USA; Penn Memory Center, University of Pennsylvania, Philadelphia, PA 19104, USA; Dementia Research Centre, UCL Queen Square Institute of Neurology, London WC1N 3BG, UK; Alzheimer Center Amsterdam, Neurology, Vrije Universiteit Amsterdam, Amsterdam UMC Location VUmc, Amsterdam 1081 HV, The Netherlands; Amsterdam Neuroscience, Neurodegeneration, Amsterdam 1081 HV, The Netherlands; Institute for Stroke and Dementia Research (ISD), University Hospital, Ludwig Maximilian University of Munich, Munich 81377, Germany; Munich Cluster for Systems Neurology (SyNergy), Munich 81377, Germany; The Sahlgrenska Academy, Institute of Neuroscience and Physiology, Psychiatry and Neurochemistry, University of Gothenburg, Gothenburg 413 90, Sweden; Alzheimer Center Amsterdam, Neurology, Vrije Universiteit Amsterdam, Amsterdam UMC Location VUmc, Amsterdam 1081 HV, The Netherlands; Amsterdam Neuroscience, Neurodegeneration, Amsterdam 1081 HV, The Netherlands; Clinical Memory Research Unit, Department of Clinical Sciences Malmö, Faculty of Medicine, Lund University, Lund 221 00, Sweden

**Keywords:** atypical Alzheimer's disease, heterogeneity, tau, connectivity, PET, fMRI

## Abstract

The link between regional tau load and clinical manifestation of Alzheimer’s disease (AD) highlights the importance of characterizing spatial tau distribution across disease variants. In typical (memory-predominant) AD, the spatial progression of tau pathology mirrors the functional connections from temporal lobe epicentres. However, given the limited spatial heterogeneity of tau in typical AD, atypical (non-amnestic-predominant) AD variants with distinct tau patterns provide a key opportunity to investigate the universality of connectivity as a scaffold for tau progression.

In this large-scale, multicentre study across 14 international sites, we included cross-sectional tau-PET data from 320 individuals with atypical AD (*n* = 139 posterior cortical atrophy/PCA-AD; *n* = 103 logopenic variant primary progressive aphasia/lvPPA-AD; *n* = 35 behavioural variant AD/bvAD; *n* = 43 corticobasal syndrome/CBS-AD), with a subset of individuals (*n* = 78) having longitudinal tau-PET data. Additionally, as an independent sample, we included regional post-mortem tau stainings from 93 atypical AD patients from two sites (*n* = 19 PCA-AD, *n* = 32 lvPPA-AD, *n* = 23 bvAD, *n* = 19 CBS-AD). Gaussian mixture modelling was used to harmonize different tau-PET tracers by transforming tau-PET standardized uptake value ratios to tau positivity probabilities (a uniform scale ranging from 0% to 100%). Using linear regression, we assessed whether brain regions with stronger resting-state functional MRI-based functional connectivity, derived from healthy elderly controls in the Alzheimer’s Disease Neuroimaging Initiative (ADNI), showed greater covariance in cross-sectional and longitudinal tau-PET and post-mortem tau pathology. Furthermore, we examined whether functional connectivity of tau-PET epicentres (i.e. the top 5% of regions with the highest baseline tau load) and tau-PET accumulation epicentres (i.e. the top 5% of regions with the highest tau accumulation rates) was associated with cross-sectional and longitudinal tau patterns.

Our findings show that tau-PET epicentres aligned with clinical variants, e.g. a visual network predominant pattern in PCA-AD (‘visual AD’) and left-hemispheric temporal predominance, particularly within the language network, in lvPPA-AD (‘language AD’). Moreover, more strongly functionally connected regions showed correlated concurrent tau-PET levels (confirmed with post-mortem data) and tau-PET accumulation rates. The functional connectivity profile of tau-PET epicentres and accumulation epicentres corresponded to tau-PET progression patterns, with higher tau-PET levels and accumulation rates in functionally close regions, and lower tau-PET levels and accumulation rates in functionally distant regions.

Our data are consistent with the hypothesis that tau propagation occurs along functional connections originating from local epicentres, across all AD clinical variants. Since tau proteinopathy is a major driver of neurodegeneration and cognitive decline, this finding may advance personalized medicine and participant-specific end points in clinical trials.

## Teaser

In a large, multicentre study using tau-PET and post-mortem data from individuals with five clinical variants of Alzheimer's disease, de Bruin *et al.* show that tau progression is not random but instead follows the brain's intrinsic functional connectivity architecture, originating from local epicentres.

**See Malotaux *et al*. (https://doi.org/10.1093/brain/awaf350) for a scientific commentary on this article.**

## Introduction

The main pathological hallmarks of Alzheimer's disease (AD) are extracellular amyloid-β (Aβ) plaques and intracellular tau neurofibrillary tangles (NFTs).^1^ Previous studies have consistently shown that compared with Aβ proteinopathy, tau proteinopathy is both spatially and temporally more strongly associated with neurodegeneration and cognitive impairment.^2-4^ This highlights the critical role of tau pathology in AD progression and the importance of better understanding how tau spreads throughout the brain.

Preclinical and human neuroimaging studies have indicated that the brain's connectome acts as a scaffold for the progression of tau across the brain.^4-26^ Specifically, tau may originate in specific local epicentres (i.e. the regions with the earliest and greatest tau burden), from where it spreads along the connections of these epicentres.^5,7,13,16-21,27^ On the other hand, functionally connected regions may also share vulnerability through commonalities in metabolic- and activity-dependent stresses, gene expression and proteostasis.^28^ These studies collectively provide robust evidence that connectivity plays a crucial role in the progression of tau pathology throughout the brain. However, previous clinical studies have primarily focused on individuals with typical AD, who present with an amnestic-predominant clinical syndrome. Although there are inter-individual differences in typical AD, the spatial heterogeneity of tau is generally limited,^7,8,19,29^ and largely adheres to the Braak staging scheme of tau pathology with a strong emphasis on medial and lateral aspects of the temporal lobe.^1,30-34^ Therefore, a crucial test of the connectivity-progression hypothesis is to determine whether connectivity-based tau progression models can be generalized to clinical phenotypes with other tau deposition patterns extending beyond the temporal lobe. Notable phenotypes of interest for this purpose are so-called atypical variants of AD, including posterior cortical atrophy (PCA: the ‘visual variant of AD’),^35^ logopenic variant primary progressive aphasia (lvPPA: the ‘language variant of AD’),^36^ behavioural variant AD (bvAD)^37^ and corticobasal syndrome (CBS: the ‘motor variant of AD’).^38^ Each of these variants shows distinct spatial tau patterns that largely correspond to the regions governing the cognitive functions that define each variant. In particular, the primary visual cortex and visual association areas show prominent tau burden in PCA; the left superior temporal gyrus in lvPPA; temporoparietal and to a lesser extent frontal regions in bvAD; and predominantly the hemisphere contralateral to the clinically affected body side, including the sensorimotor cortex, in CBS.^5,16,20,39-44^ It is, therefore, of both scientific and clinical interest to better understand the mechanisms that drive heterogeneous tau progression patterns and subsequent clinical variability in AD.

To test whether functional connectivity is a universal predictor of tau progression, independent of clinical phenotype, we aimed to assess tau-PET progression patterns as well as post-mortem tau distributions in Aβ-positive individuals with PCA, lvPPA, bvAD, CBS and typical AD. Because atypical AD variants are relatively rare,^45,46^ we conducted a large-scale multicentre study on atypical AD, using patient-specific tau-PET across 14 sites worldwide (*n* = 320, with *n* = 68 typical AD cases serving as the benchmark group) and resting-state functional MRI (fMRI) data from healthy elderly controls in the Alzheimer's Disease Neuroimaging Initiative [ADNI, *n* = 42 cognitively normal (CN) Aβ-negative individuals], as well as post-mortem datasets from two sites [University of Pennsylvania (UPENN) *n* = 63, University of California, San Francisco (UCSF) *n* = 30]. Our objectives were to: (i) assess the spatial heterogeneity of tau-PET distribution and identify subject-level tau-PET epicentres across AD variants; (ii) test whether brain regions with stronger functional connectivity to each other show greater covariance in cross-sectional tau-PET and gold standard post-mortem tau pathology; (iii) assess whether functional connectivity of subject-level tau-PET epicentres (i.e. regions with the highest tau at baseline) predicts cross-sectional tau patterns; (iv) test whether brain regions with stronger functional connectivity to each other show greater covariance in tau-PET accumulation rates; and (v) establish whether functional connectivity of subject-level tau-PET accumulation epicentres (i.e. regions with the highest accumulation of tau over time) predicts longitudinal tau progression sequences. We hypothesize that, in all clinical variants of AD, tau propagates along functional connections of the subject-level tau-PET epicentre (i.e. the regions with the highest tau-PET level or fastest tau-PET accumulation), indicating a universal scaffold for the progression of tau pathology. In addition, we hypothesize that brain regions with stronger functional connectivity to each other show stronger covariance in tau pathology on post-mortem examination. By investigating the association between tau and functional connectivity this study aims to deepen the understanding of mechanisms of tau progression and AD heterogeneity, potentially informing more targeted therapeutic strategies and tailored clinical trial end points.

## Materials and methods

### Tau-PET sample

We included individuals with AD recruited at 14 international sites [Amsterdam, Cambridge, Cologne, Leipzig, Lund, Mayo, Massachusetts General Hospital (MGH), McGill, Munich, University College London (UCL), UCSF, UPENN, Washington and Yale]. Inclusion criteria were a clinical diagnosis of atypical AD according to the contemporaneously available criteria,^35-38^ confirmed positive Aβ status (either via PET or CSF), and the availability of at least one tau-PET scan and basic demographic and clinical information. We also received data from several sites on individuals with typical AD, whom we decided to include as a reference group. Longitudinal tau-PET data was available for a subset of individuals within each AD variant. However, due to the relatively small sample sizes in the other variants, we only included this data for PCA-AD and lvPPA-AD. In addition to data from AD patients, we also obtained data from control subjects (primarily consisting of CN Aβ-negative individuals) scanned using the same tau-PET tracers. This ensured the inclusion of varying tau-PET levels at both the lower and higher ends of the spectrum, enabling Gaussian mixture modelling-based transformation of tau-PET standardized uptake value ratio (SUVR) values to tau positivity probabilities^7^ (see Supplementary material, ‘Methods’ section). Besides control data from the aforementioned 14 cohorts, we also included control data from ADNI for this. All study procedures were conducted in accordance with the Declaration of Helsinki, ethical approval was obtained by investigators at each site. All study participants provided written informed consent.

### Neuroimaging data collection

Every individual underwent at least one tau-PET scan following site-specific acquisition protocols.^4,14,16,47-57^ Both scanning protocols and specific tracers varied by site, with tracers including ^18^F-flortaucipir, ^18^F-MK6240, ^18^F-PI2620 and ^18^F-RO948. Before data preprocessing, all imaging data were reviewed for artefacts and image quality.

Preprocessing steps and region-of-interest (ROI) extraction were performed either centrally (in Amsterdam/Munich) or locally. Collaborators from Cambridge, Cologne, Lund, Mayo, MGH, McGill, UCL, UCSF, UPENN, and Washington conducted both preprocessing and ROI extraction at their respective sites.^4,14,16,48,50-52,54-56^ PET scans from Leipzig, Yale, Amsterdam and Munich were centrally analysed.^47,49^ To ensure consistency across all sites, ROI extraction followed a standardized protocol: each site was provided with an R script, the Schaefer parcellation and a grey matter mask, which were used to generate the Schaefer 200 values: https://github.com/OssenKoppeLab/HdeBruin_Atypical_AD.

For preprocessing, individual PET scan frames were realigned and averaged to create average images, which were subsequently co-registered to the corresponding T1-weighted MRI. For ROI extraction, the cortical Schaefer atlas^58^ with 200 ROIs applied to the structural MRI image was used to extract regional tau-PET SUVR data, which was adjusted to an inferior cerebellar grey reference region.

### Assessment of tau-PET change

We assessed tau-PET change over time by computing the annual SUVR change for every individual across 200 ROIs. For each individual and each ROI, we fitted a separate linear model defined as: tau-PET SUVR = β_0_ + β_1_ × time + ε, where tau-PET SUVR reflects the tau load at a given time point, β_0_ is the intercept, β_1_ is the slope, time is the follow-up time in years and ε is the residual error term. To express the change as a relative percentage, we normalized each ROI's slope (β_1_) by the individual's baseline SUVR for that ROI (i.e. at follow-up time = 0): relative tau-PET change (% per year) = (β_1_/tau-PET SUVR_0_) × 100. This yielded the annual percentage change in tau-PET signal per ROI.

### Identification of tau-PET epicentres

To determine the tau-PET epicentre for each individual, we employed a previously established method^7,19,59^ that is based on the premise that brain regions showing early abnormal tau deposition would display higher tau levels than regions with later abnormal tau deposition. At the subject level, we arranged all Schaefer ROIs according to their tau-PET SUVR values at baseline, thereby delineating the estimated cross-sectional sequence of tau propagation. Subject-specific tau epicentres were then defined as the top 5% of ROIs (i.e. 10 ROIs in total) with the highest tau-PET SUVR values. We determined tau-PET accumulation epicentres in a similar way, see Supplementary material, ‘Methods’ section.

### Post-mortem sample

As an independent sample (i.e. not the same individuals as in the tau-PET sample), we recruited data from AD cases who had undergone post-mortem examination at two sites (UPENN and UCSF).^60,61^ Inclusion criteria were an ante-mortem clinical diagnosis of atypical AD, AD being the primary neuropathological diagnosis, the availability of post-mortem ordinal or quantitative tau assessments in preferably ∼10 probe extraction sites per individual including their anatomical labelling, and the presence of basic demographic and ante-mortem clinical information.

### Post-mortem assessment

Post-mortem data collection and preparation followed standard pre-established procedures.^60,61^ All assessments were unilateral (see Supplementary methods). UPENN tau assessments included ordinal ratings (0 = none, 1 = mild, 2 = moderate, 3 = severe) of paired helical filament-1 (PHF-1) staining^62^ across 19 ROIs (i.e. the amygdala, dentate gyrus, CA1/subiculum, entorhinal cortex, middle frontal cortex, angular gyrus, superior/middle temporal cortex, anterior cingulate, occipital cortex, caudate/putamen, globus pallidus, thalamus/subthalamic nucleus, midbrain, substantia nigra, pons, locus coeruleus, medulla, cerebellum and sensory cortex). From these ROIs, we selected those with sufficient data and relevance for assessing functional connectivity for our fMRI-based analyses. Additionally, due to the small size of individual hippocampal regions, we combined these into a single hippocampal ROI. This selection narrowed the 19 ROIs down to 9: the amygdala, hippocampus, entorhinal cortex, middle frontal cortex, angular gyrus, superior/middle temporal cortex, anterior cingulate, occipital cortex, thalamus/subthalamic nucleus. UCSF tau assessments consisted of quantitative thioflavin-S fluorescent microscopy staining^61^ across six ROIs (i.e. the CA1, subiculum, middle frontal gyrus, angular gyrus, superior temporal gyrus and primary motor cortex), resulting in a density score (i.e. the number of NFTs per mm^2^) per ROI. All six ROIs had sufficient data and were fMRI compatible. We again combined the individual hippocampal regions into a single hippocampal ROI, resulting in five total ROIs. For both samples, we used known cortical and subcortical brain atlases [i.e. the automated anatomical labelling (AAL) atlas, computational brain anatomy laboratory (CoBrA) atlas, Julich atlas and Neuromorphometrics atlas]^63-66^ to create an MRI brain atlas based on the selected ROIs.^67^ Although pathology assessments were unilateral, ROIs were mapped bilaterally in the atlas to align with the functional connectivity analyses.

### Assessment of covariance in tau-PET and covariance in post-mortem tau

Cross-sectional and longitudinal tau-PET covariance were defined as AD variant-average Fisher z-transformed partial Pearson correlations between, respectively, tau positivity probabilities or tau-PET SUVR percentage change rates of all 200 Schaefer ROI pairs, while adjusting for age, sex, site (and Euclidean distance). We assessed tau-PET covariance both across the whole brain and in seven individual canonical resting-state fMRI networks.^68^ Post-mortem tau covariance was determined by calculating Fisher z-transformed partial Spearman (UPENN) and Pearson (UCSF) correlations between post-mortem tau semi-quantitative and quantitative ratings of all ROI pairs (based on the created bilateral MRI brain atlas), respectively, while adjusting for age and sex.

### Functional connectivity assessment

We utilized resting-state fMRI data from an independent sample of 42 CN Aβ-negative individuals from ADNI to construct the connectome template, along which tau progression was modelled. Pre-processing of the fMRI data involved several steps, starting with slice-timing correction and motion correction, with all volumes realigned to the first volume. Echo planar imaging (EPI) images were subsequently co-registered to their respective T1-weighted structural scans. Grey matter, eroded white matter and eroded CSF segments derived from the T1 images were transformed into EPI space based on rigid transformation parameters.

To denoise the fMRI data, we regressed out nuisance signals, including time series from eroded white matter and CSF, as well as six motion parameters. Additional steps included detrending and band-pass filtering within the 0.01–0.08 Hz frequency range, with all processing conducted in native EPI space. To minimize the impact of motion artefacts on connectivity estimates, motion scrubbing was performed. Volumes with frame-wise displacement above 0.5 mm, along with one preceding and two following volumes, were excluded. Each participant retained at least 5 min of usable resting-state fMRI data after scrubbing. Spatial smoothing was avoided to prevent artificial enhancement of connectivity signals due to spatial overlap (and thus, spilling) between neighbouring brain regions. Finally, the pre-processed resting-state fMRI data were warped to Montreal Neurological Institute (MNI) space using spatial normalization parameters from CAT12.

Subsequently, for the tau-PET part of this study, we applied the 200 ROI Schaefer atlas to the fMRI data to generate a functional connectivity matrix representing Fisher z-transformed Pearson correlations between fMRI time series [i.e. fluctuations in the blood oxygen level-dependent (BOLD) signal] of all possible ROI pairs. Based on a previously established method, this matrix was density thresholded at 30% (i.e. 30% of the strongest positive connections were retained) and transformed to functional connectivity-based distance.^7^ The distance metric reflects the shortest functional path length between each ROI pair, where regions with stronger direct or indirect connections are considered closer together, and regions with weaker or no connections are considered more distant. Besides assessing functional connectivity across the whole brain, we also determined functional connectivity in the same seven canonical resting-state fMRI networks (i.e. the default mode network, dorsal and ventral attention network, frontoparietal control network, limbic network, motor network and visual network) that were used for assessing tau-PET covariance.^68^ For the post-mortem part of this study, we applied our MRI brain atlas to the fMRI data to generate a functional connectivity matrix representing Fisher z-transformed Pearson correlations between the fMRI time series of all possible ROI pairs. We did not transform functional connectivity to functional connectivity-based distance for our post-mortem analyses due to sparsity and limited adjacency of regions.

### Statistical analyses

Group differences in baseline demographics were assessed using ANOVA or Kruskal–Wallis tests for continuous variables and chi-squared tests of independence for categorical variables. In the case of cell counts <5, Monte Carlo simulations with 20 000 replications (*B* = 20 000) were employed to estimate the *P*-values for the chi-squared tests. If a statistically significant main effect was observed, Tukey's Honestly Significant Difference (HSD) test was used as a *post hoc* test following ANOVA, Dunn's test following the Kruskal–Wallis test, and Fisher's exact tests following chi-square tests. Dunn's test and Fisher's exact tests were adjusted for multiple comparisons using the Bonferroni correction. When data was missing for a category [education *n* = 89, Apolipoprotein E (*APOE*ε4) status *n* = 150, Mini-Mental State Examination (MMSE) *n* = 79], individuals were excluded from that specific analysis. For our cross-sectional *in vivo* covariance analyses, the association between inter-regional functional connectivity-based distance and age-, sex- and site-adjusted inter-regional tau-PET covariance was assessed using linear regression for each AD variant, both in the whole brain and in the seven individual resting-state fMRI networks. As a sensitivity analysis, we repeated these analyses with additional adjustment of tau-PET covariance for inter-regional Euclidean distance (i.e. the geometric distance between the centre of mass of each ROI). Furthermore, to test the robustness of our findings, we performed a previously described bootstrapping procedure.^8^ In this procedure, 1000 different connectivity null models were generated by shuffling the 200 × 200 connectivity matrix while preserving the weight and degree distribution. Subsequently, we re-ran the whole-brain linear model 1000 times, each time using a different connectivity matrix from the set of 1000 null models. This procedure resulted in a distribution of 1000 *β*-values based on the null models, which were compared against the actual *β*-value using exact tests. For our post-mortem covariance analyses, the association between inter-regional functional connectivity and age- and sex-adjusted inter-regional tau pathology covariance was also assessed using linear regression. Here, we pooled the data from all atypical AD variants to increase statistical power. To study cross-sectional *in vivo* tau progression, at the subject level, linear regression was used to assess the association between functional connectivity-based distance to the tau epicentre and tau-PET SUVR, after which *β*-values were visualized per AD variant. Additionally, we grouped all non-epicentre regions into quartiles based on their functional proximity to the epicentre (quartile 1 = close, quartile 4 = distant) and compared tau positivity probabilities across quartiles using paired Wilcoxon signed-rank tests. For our longitudinal *in vivo* covariance analyses, we assessed the association between inter-regional functional connectivity-based distance and inter-regional tau-PET annual percentage change covariance through linear regression for each AD variant, both across the whole brain and in the seven individual resting-state fMRI networks. We applied the same bootstrapping procedure used in our cross-sectional covariance analyses to evaluate the robustness of our findings. To study longitudinal *in vivo* tau progression, at the subject level, linear regression was utilized to assess the association between functional connectivity-based distance to the tau accumulation epicentre and tau-PET annual percentage change, after which *β*-values were visualized per AD variant. Moreover, we grouped all non-accumulation-epicentre regions into quartiles based on their functional proximity to the accumulation epicentre (quartile 1 = close, quartile 4 = distant) and compared tau-PET percentage change rates across quartiles using paired Wilcoxon signed-rank tests. Significance for all effects was determined at a two-tailed *α* = 0.05. All statistical analyses were performed using R statistical software (R Foundation for Statistical Computing, Vienna, Austria). Brain surface renderings were generated using the Connectome Workbench.

## Results

### Sample characteristics

For the tau-PET section of this study, we included 388 Aβ-positive (either on PET or CSF) individuals with a clinical diagnosis of AD. For two individuals, Aβ status was not known, but they were included based on a positive tau-PET scan. In total, 139 individuals were classified as PCA-AD, 103 as lvPPA-AD, 35 as bvAD, 43 as CBS-AD and 68 as typical (or amnestic) AD. Baseline tau-PET data was available for all 388 individuals. Longitudinal tau-PET data was available for 78 individuals with PCA-AD or lvPPA-AD (PCA-AD *n* = 45, mean follow-up time: 1.40 ± 0.63 years, range: 0.77–3.16 years; lvPPA-AD *n* = 33, mean follow-up time: 1.41 ± 0.78 years, range: 0.79–4.07 years). For the post-mortem section of this study, we included 93 individuals with an ante-mortem clinical diagnosis of atypical AD as well as post-mortem neuropathologically confirmed AD from two sites. One of these sites (UPENN) provided a larger semi-quantitative dataset (*n* = 63; 12 PCA-AD, 23 lvPPA-AD, 13 bvAD and 15 CBS-AD), while the other site (UCSF) contributed a smaller quantitative dataset (*n* = 30; 7 PCA-AD, 9 lvPPA-AD, 10 bvAD and 4 CBS-AD), which was used as a replication sample. Baseline demographic, clinical and imaging/neuropathological information across AD variants is presented in Table 1 (tau-PET cohort) and Table 2 (post-mortem cohort), while baseline demographic and clinical information across all sites can be found in Supplementary Table 1.

**Table 1:** Tau-PET cohort: demographic, clinical and imaging information across atypical AD variants

	PCA-AD	lvPPA-AD	bvAD	CBS-AD	Typical AD	Total	*P*
*N*	139	103	35	43	68	388	
Age (years)^a^	64.05 ± 7.66^b,c,d^	67.75 ± 8.31^d,e^	65.95 ± 8.05^c,d^	71.76 ± 8.56^e,f^	71.77 ± 9.25^b,e,f^	67.41 ± 8.79	<0.001
Female^g^	85 (61.2)	52 (50.5)	18 (51.4)	23 (53.5)	32 (47.1)	210 (54.1)	0.310
Education (years)^a^	15.43 ± 2.99	15.58 ± 3.38^d^	14.68 ± 3.76	14.58 ± 3.49	13.91 ± 3.58^b^	15.10 ± 3.36	0.045
*APOE*ε4 carrier^g^	44 (50.0)	30 (44.8)	21 (65.6)	6 (60.0)	26 (63.4)	127 (53.4)	0.194
MMSE^h^	21.45 ± 5.64^c,d^	21.49 ± 5.61	20.85 ± 5.80^c^	24.04 ± 5.78^e,f^	23.88 ± 4.50^e^	22.01 ± 5.56	0.003
Tau-PET tracer^g^							<0.001^i^
^18^F-flortaucipir	96 (69.1)	73 (70.9)	15 (42.9)	18 (41.9)	18 (26.5)	220 (56.7)	
^18^F-MK6240	25 (18.0)	18 (17.5)	18 (51.4)	0 (0.0)	29 (42.6)	90 (23.2)	
^18^F-PI2620	12 (8.6)	7 (6.8)	0 (0.0)	24 (55.8)	21 (30.9)	64 (16.5)	
^18^F-RO948	6 (4.3)	5 (4.9)	2 (5.7)	1 (2.3)	0 (0.0)	14 (3.6)	

Values are mean ± standard deviation for continuous variables and *n* (%) for categorical variables. Differences between groups were assessed using ANOVA, chi-squared tests of independence or the Kruskal-Wallis test. In the case of cell counts <5, Monte Carlo simulations with 20 000 replications (*B* = 20 000) were employed to estimate the *P*-values for the chi-squared tests. If a statistically significant main effect was observed, Tukey’s Honestly Significant Difference (HSD) test was used as a *post hoc* test following ANOVA, Fisher’s exact tests following chi-square tests, and Dunn’s test following the Kruskal-Wallis test. Fisher’s exact tests and Dunn’s test were adjusted for multiple comparisons using the Bonferroni correction. When data was missing for a category (education *n* = 89, *APOE*ε4 status *n* = 150, MMSE *n* = 79), individuals were excluded from that specific analysis. AD = Alzheimer’s disease; *APOE* = apolipoprotein E; bvAD = behavioural variant Alzheimer’s disease; CBS = corticobasal syndrome; lvPPA = logopenic variant primary progressive aphasia; MMSE = mini-mental state examination; PCA = posterior cortical atrophy.

^a^Differences between groups were assessed using ANOVA.

^b^Significantly different from lvPPA-AD.

^c^Significantly different from CBS-AD.

^d^Significantly different from typical AD.

^e^Significantly different from PCA-AD.

^f^Significantly different from bvAD.

^g^Differences between groups were assessed using chi-squared tests of independence.

^h^Differences between groups were assessed using the Kruskal-Wallis test.

^i^All group comparisons were significant, except for PCA-AD versus lvPPA-AD.

**Table 2:** Post-mortem cohort: demographic, clinical and neuropathological information across atypical AD variants per site

	UPENN				UCSF			
	PCA-AD	lvPPA-AD	bvAD	CBS-AD	PCA-AD	lvPPA-AD	bvAD	CBS-AD
*N*	12	23	13	15	7	9	10	4
Age at death, years	68.08 ± 8.97	69.26 ± 7.92	74.00 ± 10.88	65.87 ± 7.46	63.29 ± 3.64	66.56 ± 5.81	63.50 ± 6.98	74.75 ± 9.25
Female	3 (25.0)	10 (43.5)	5 (38.5)	11 (73.3)	6 (85.7)	6 (66.7)	2 (20.0)	2 (50.0)
Education, years	15.83 ± 3.10	16.82 ± 2.79	15.00 ± 3.11	15.14 ± 2.41	15.43 ± 2.15	14.89 ± 1.96	16.12 ± 2.75	18.00 ± 6.32
MMSE	13.42 ± 8.58	9.81 ± 7.53	13.73 ± 8.30	9.14 ± 4.79	12.17 ± 12.42	7.22 ± 5.49	13.11 ± 6.79	19.75 ± 9.50
A (Aβ plaques)								
2	0 (0.0)	0 (0.0)	0 (0.0)	1 (6.7)	0 (0.0)	0 (0.0)	0 (0.0)	1 (25.0)
3	12 (100.0)	23 (100.0)	13 (100.0)	14 (93.3)	4 (100.0)	7 (100.0)	9 (100.0)	3 (75.0)
B (NFTs)								
2	0 (0.0)	0 (0.0)	0 (0.0)	1 (6.7)	0 (0.0)	0 (0.0)	0 (0.0)	1 (25.0)
3	12 (100.0)	23 (100.0)	13 (100.0)	14 (93.3)	7 (100.0)	9 (100.0)	10 (100.0)	3 (75.0)
C (NPs)								
2	2 (16.7)	0 (0.0)	2 (15.4)	2 (13.3)	0 (0.0)	0 (0.0)	0 (0.0)	0 (0.0)
3	10 (83.3)	23 (100.0)	11 (84.6)	13 (86.7)	7 (100.0)	9 (100.0)	10 (100.0)	4 (100.0)

Values are mean ± standard deviation for continuous variables and *n* (%) for categorical variables. Aβ plaques, NFTs and NPs are presented according to ABC-score criteria (A = Aβ plaques using the Thal phase system; B = NFTs using the Braak staging system; C = NPs based on the CERAD criteria).^118^  *N* = 4 missing for education (*n* = 2 UPENN, *n* = 2 UCSF), *n* = 7 for MMSE (*n* = 5 UPENN, *n* = 2 UCSF), *n* = 6 for A (Aβ plaques; all UCSF). Aβ = amyloid-β; AD = Alzheimer’s disease; bvAD = behavioural variant Alzheimer’s disease; CBS = corticobasal syndrome; lvPPA = logopenic variant primary progressive aphasia; MMSE = mini-mental state examination; NFTs = neurofibrillary tangles; NPs = neuritic plaques; PCA = posterior cortical atrophy; UCSF = university of California, San Francisco; UPENN = university of Pennsylvania.

### Tau-PET spatial distribution and epicentres align with clinical phenotypes

**Figure 1:**
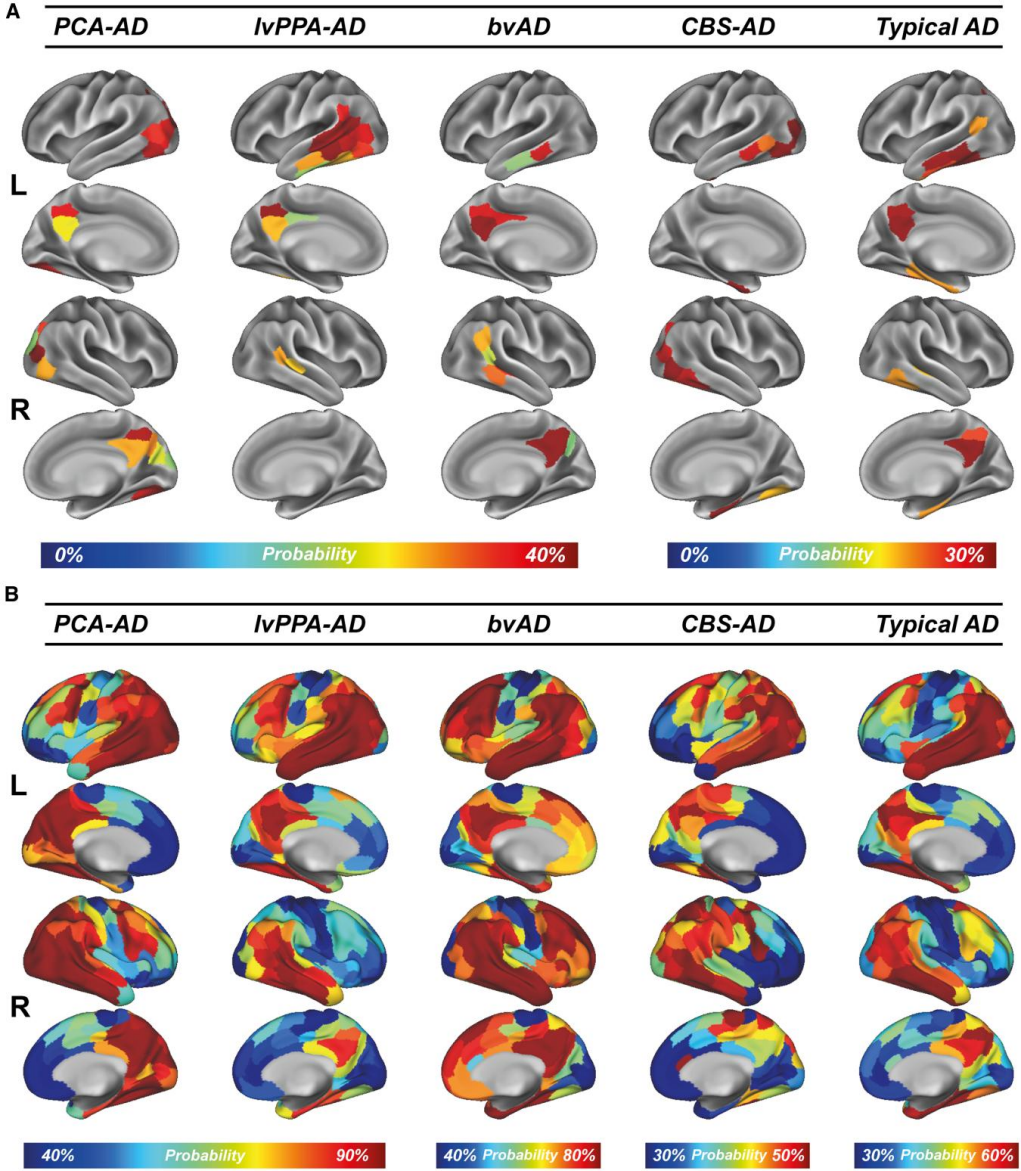
**Tau-PET epicentres and positivity across AD variants.** Tau epicentres (i.e. the regions with the assumed earliest and greatest tau burden) were defined at the subject level as the 5% regions with the highest tau-PET SUVRs at baseline. Group-average epicentre probabilities (**A**) indicate the likelihood of a region being part of the epicentre, with only epicentre probabilities ≥20% shown. Group-average baseline tau-PET positivity probabilities (a uniform tau-PET scale ranging from 0% to 100%) across AD variants are shown in (**B**). AD = Alzheimer’s disease; bvAD = behavioural variant Alzheimer’s disease; CBS = corticobasal syndrome; L = left; lvPPA = logopenic variant primary progressive aphasia; PCA = posterior cortical atrophy; R = right; SUVR = standardized uptake value ratio.

Our first objective was to assess the heterogeneity of tau-PET distribution and identify tau-PET epicentres across AD variants. Accordingly, similar to previous approaches,^7,19^ and pending the development of widely accepted tau-PET harmonization methods,^69^ we harmonized the different tau-PET tracers from the different sites by transforming tau-PET SUVRs to tau positivity probabilities (0% to 100%) using Gaussian mixture modelling. For each AD variant, we computed tau-PET positivity probabilities in 200 Schaefer atlas^58^ ROIs (Fig. 1B). The tau positivity probability maps generally resembled the previously described topography of each clinical variant, including a posterior pattern in PCA-AD,^16,35,40,70,71^ pronounced left-hemispheric temporal involvement in lvPPA-AD,^16^  ^,36,40,72,73^ a diffuse pattern primarily involving the temporoparietal regions, with additional frontal involvement, in bvAD,^16,37,47^ and bilateral temporoparietal predominance in typical AD.^1,16,40^ The exception was the CBS-AD group, which showed a prominent posterior pattern (Supplementary Fig. 1B shows the tau positivity probabilities for individuals with CBS-AD grouped by the predominant clinically affected side). Although there was some unique involvement of the sensorimotor cortex (that was not observed in any of the other variants), this was less pronounced than in several previous studies.^40,42,74,75^ Regional tau-PET positivity probabilities were generally lower in CBS-AD and typical AD compared with the other variants. Furthermore, in line with the spatial distribution of tau positivity probabilities, tau epicentres (i.e. the top 5% of regions exhibiting the highest baseline tau-PET SUVR, determined at the subject level) were highly heterogeneous across variants (Fig. 1A and Supplementary Fig. 1A). Correlations between the entire multisite cohort and the average of subsets of the data derived by systematically excluding one site at a time (i.e. a leave-one-site-out approach) were near perfect across all AD variants (Supplementary Fig. 2). In addition, Levene's test showed that there were no variance differences across all leave-one-site-out scenarios (all *P* > 0.05; see Supplementary Table 2). These findings support the robustness of our results and demonstrate the validity of the tau positivity probability approach, prompting us to continue using it in our main (group-level) analyses.

### Regions with stronger functional connectivity exhibit greater *in vivo* and post-mortem tau covariance

**Figure 2:**
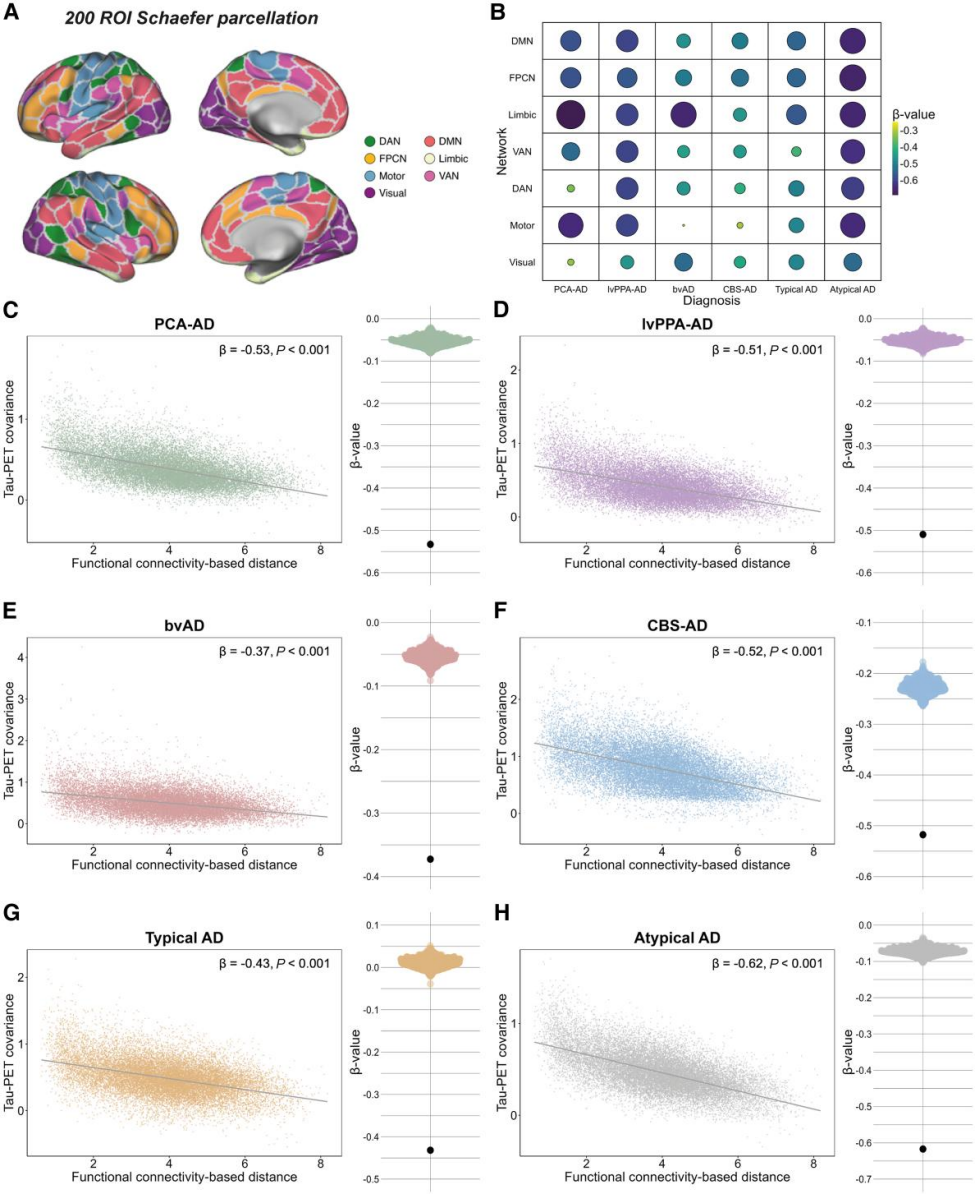
**Association between functional connectivity and covariance in tau-PET across variants of AD.** Surface rendering of the 200 ROI brain atlas used for tau-PET and resting-state functional MRI (fMRI) data in ROI-based analyses (**A**). Functional connectivity was defined as Fisher z-transformed Pearson correlations between fluctuations in the BOLD signal of all possible 200 Schaefer ROI pairs in 42 CN Aβ-negative individuals from ADNI. The 200 × 200 ROI functional connectivity matrix was density thresholded at 30% (i.e. 30% of the strongest positive connections were retained) and transformed to functional connectivity-based distance (strongly connected regions are ‘close’, while weakly or indirectly connected regions are ‘distant’). Tau-PET covariance was defined as AD variant-average Fisher z-transformed partial Pearson correlations between tau positivity probabilities of all possible ROI pairs, while adjusting for age, sex and site. The association between inter-regional functional connectivity-based distance and inter-regional tau-PET covariance was assessed using linear regression for all AD variants, both across the whole brain (**C**–**H**) and in seven individual resting-state fMRI networks separately (**A** and **B**). To test the robustness of these findings, we re-ran the whole-brain analysis 1000 times, each time using a different connectivity null model from the set of 1000 null models that were generated by shuffling the connectivity matrix while preserving the weight and degree distribution. This procedure resulted in a distribution of *β*-values based on the null models, as depicted in the beeswarm panels in **C**–**H**, where the actual *β*-value (furthest data-point) always exceeded the null model *β*-values. Aβ = amyloid-β; AD = Alzheimer’s disease; ADNI = Alzheimer’s disease neuroimaging initiative; BOLD = blood oxygen level-dependent; bvAD = behavioural variant Alzheimer’s disease; CBS = corticobasal syndrome; CN = cognitively normal; DAN = dorsal attention network; DMN = default mode network; FPCN = frontoparietal control network; lvPPA = logopenic variant primary progressive aphasia; PCA = posterior cortical atrophy; ROI = region of interest; VAN = ventral attention network.

Our second objective was to test whether higher inter-regional functional connectivity is associated with higher covariance in cross-sectional tau-PET uptake and post-mortem tau pathology. To investigate this, we first explored the association between inter-regional functional connectivity-based distance and inter-regional covariance in tau-PET through linear regression. In all AD variants, analysed separately, greater AD variant-average tau-PET covariance was associated with shorter functional connectivity-based distance. We observed this relationship when assessing connectivity and tau covariance across the whole brain (Fig. 2C–H, PCA-AD: *β* = −0.53, *P* < 0.001, lvPPA-AD: *β* = −0.51, *P* < 0.001, bvAD: *β* = −0.37, *P* < 0.001, CBS-AD: *β* = −0.52, *P* < 0.001, typical AD: *β* = −0.43, *P* < 0.001, atypical AD altogether: *β* = −0.62, *P* < 0.001), as well as within seven individual functional brain networks (Fig. 2 A and B and Supplementary Fig. 3), suggesting that the association between connectivity and covariance in tau is not confined to specific high-tau regions. The results remained consistent when adjusting for inter-regional Euclidean distance (Supplementary Fig. 4), suggesting that functional connectivity (and not spatial proximity) is the main driver of these associations. To test the robustness of these findings, we performed a previously described bootstrapping procedure in which 1000 connectivity null models were generated by shuffling the 200 × 200 connectivity matrix while preserving the weight and degree distribution.^8^ Subsequently, we re-ran the whole-brain linear model 1000 times, each time using a different connectivity matrix, which resulted in a distribution of null-model *β*-values (Fig. 2C–H). We then compared the actual *β*-value from the observed true connectivity matrix to the *β*-values generated by the null models using exact tests. This enabled us to determine the frequency with which the *β*-values from the null models exceeded the actual *β*-value. For all AD variants, the null model *β*-values never exceeded the actual *β*-value, further strengthening our findings.

**Figure 3:**
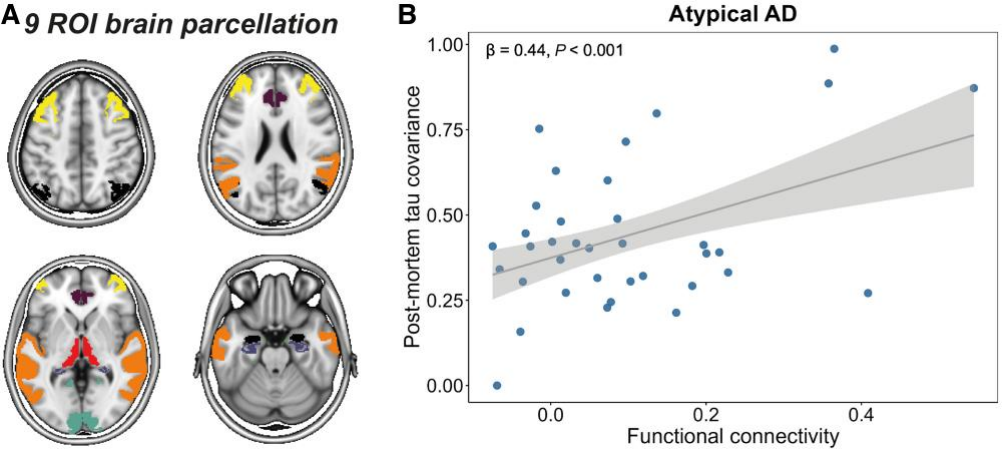
**Association between functional connectivity and covariance in post-mortem tau pathology in atypical AD.** Using established cortical and subcortical brain atlases (i.e. AAL, CoBrA, Julich, Neuromorphometrics), we created a bilateral MRI brain atlas for the regions with post-mortem tau assessment (*n* = 9, see **A**). Functional connectivity was defined as Fisher z-transformed Pearson correlations between functional MRI (fMRI) time series (reflective of fluctuations in the BOLD signal) of all ROI pairs in 42 CN Aβ-negative individuals from ADNI. Tau covariance was defined as Fisher z-transformed partial Spearman correlations between semi-quantitative tau pathology ratings of all ROI pairs, while adjusting for age and sex. We pooled the data from all AD variants to increase statistical power. The association between inter-regional functional connectivity and inter-regional tau pathology covariance was assessed using linear regression (**B**). AAL = automated anatomical labelling; Aβ = amyloid-β; AD = Alzheimer’s disease; ADNI = Alzheimer’s disease neuroimaging initiative; BOLD = blood oxygen level-dependent; CN = cognitively normal; CoBrA = computational brain anatomy laboratory; ROI = region of interest.

In addition to the tau-PET analyses, we also examined the relationship between functional connectivity and tau covariance using post-mortem data. Given that the tau-PET signal can be confounded by factors other than tau pathology, such as binding to off-target sources like astrogliosis or iron accumulation,^76,77^ the reliability of the tau-PET findings would be strengthened by post-mortem replication. We assessed the association between inter-regional functional connectivity [i.e. a matrix with Fisher z-transformed Pearson correlations between the fMRI time series of all ROI pairs (using ADNI elderly control data)] and inter-regional post-mortem tau covariance [i.e. Fisher z-transformed age- and sex-adjusted partial Spearman (in the semi-quantitative UPENN dataset) or Pearson (in the quantitative UCSF dataset) correlations between tau pathology ratings of all ROI pairs] using linear regression. For this analysis, we pooled data from all atypical AD variants to increase statistical power. Consistent with our hypothesis and previous tau-PET results, UPENN data (*n* = 63) showed that stronger functional connectivity was associated with higher covariance in post-mortem tau pathology across nine ROIs, *β* = 0.44, *P* < 0.001 (Fig. 3). Although the analysis did not reach statistical significance, the direction of this effect was confirmed in the smaller UCSF replication sample (*n* = 30), *β* = 0.36, *P* = 0.116 (Supplementary Fig. 5).

### Regions more functionally connected to the tau-PET epicentre show higher tau-PET levels

**Figure 4:**
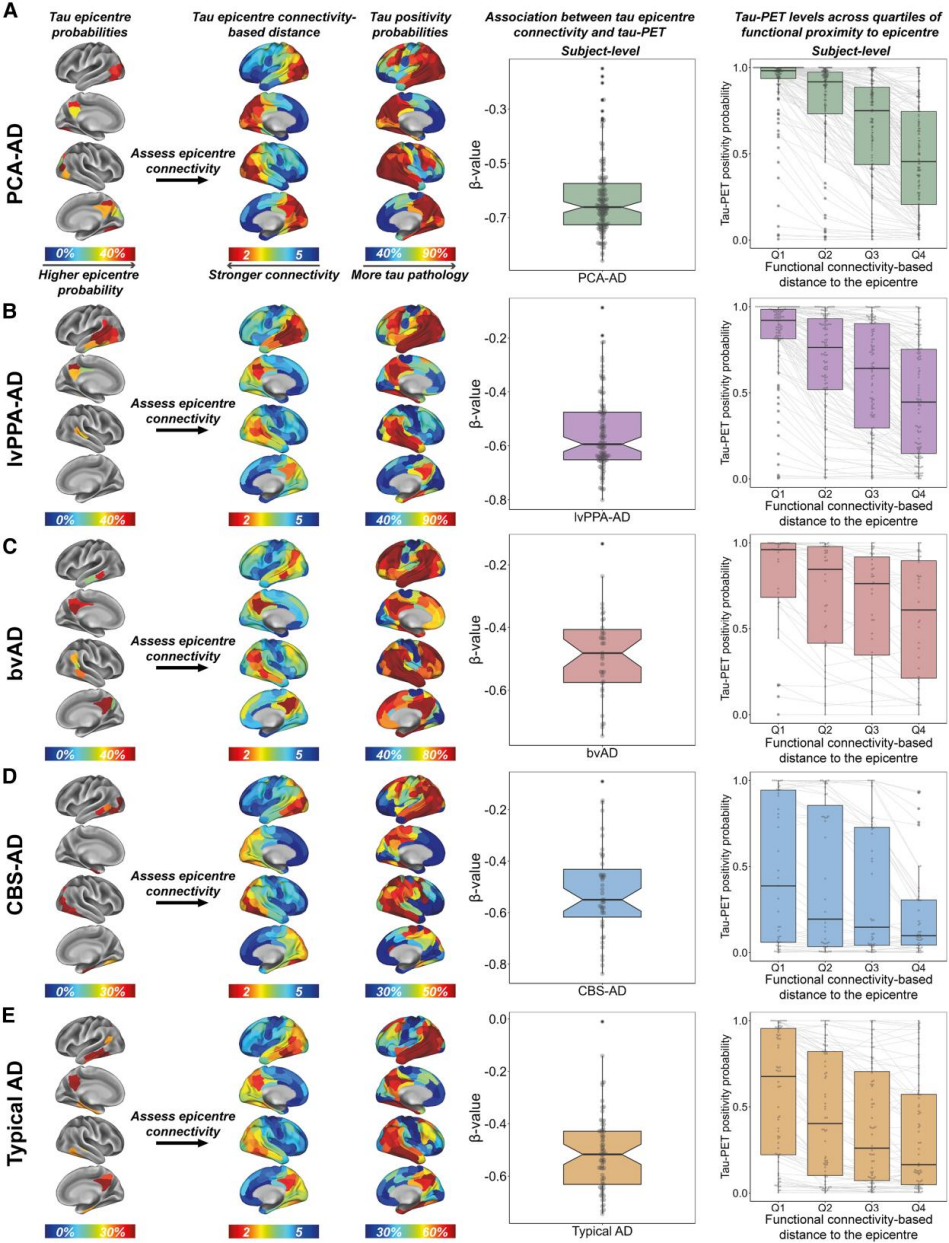
**Association between tau epicentre connectivity and tau-PET across AD variants.** Tau epicentre connectivity was determined by taking the functional connectivity-based distance (see Fig. 2 for method specifications) of each non-epicentre ROI (*n* = 190) to the epicentre (*n* = 10). For each individual, linear regression was used to assess the association between functional connectivity-based distance to the tau epicentre and tau-PET SUVR. Subject-level *β*-values are visualized per AD variant in the notched boxplots in **A**–**E**. Additionally, all non-epicentre regions were grouped into quartiles based on their functional proximity to the epicentre (quartile 1 = shortest functional connectivity-based distance, quartile 4 = longest functional connectivity-based distance), and tau positivity probabilities across quartiles were compared using paired Wilcoxon signed-rank tests. AD = Alzheimer’s disease; bvAD = behavioural variant Alzheimer’s disease; CBS = corticobasal syndrome; lvPPA = logopenic variant primary progressive aphasia; PCA = posterior cortical atrophy; Q = quartile; ROI = region of interest; SUVR = standardized uptake value ratio.

Our third objective was to test the hypothesis that functional connectivity of specific tau-PET epicentres is associated with tau progression sequences inferred from cross-sectional data. For each AD variant separately, we used linear regression to test the association between epicentre connectivity and tau-PET levels. Our findings showed that, across all AD variants, a shorter functional connectivity-based distance to the tau epicentre was associated with higher tau-PET SUVRs, both when tested at the subject level (Fig. 4A–E) and per tracer at the group level (Supplementary Fig. 6). To further investigate this, we divided all brain regions (excluding the epicentre) into quartiles based on their functional proximity to the tau epicentre. We then examined whether regions in the lower quartiles (i.e. regions functionally most strongly connected to the tau epicentre) had higher tau positivity probabilities than those in the higher quartiles (i.e. regions functionally distant from the tau epicentre). As expected, a gradient of increasing tau positivity probabilities was observed from quartile 4 to quartile 1 across all AD variants (Fig. 4A–E; paired Wilcoxon signed-rank tests indicating *P* < 0.05 for all quartile comparisons). When repeating these analyses in a subset of individuals with subject-level fMRI available (PCA-AD *n* = 6, lvPPA-AD *n* = 5, CBS-AD *n* = 5), the results were generally comparable to the main analyses (Supplementary Fig. 7). Paired Wilcoxon signed-rank tests for the entire atypical AD group were significant (*P* < 0.05) for all quartile comparisons, except for quartile 2 versus quartile 3, which was borderline significant (*P* = 0.07). These analyses support our hypothesis that tau progression across the brain follows the pattern of functional connections from the tau epicentre, across atypical AD variants.

### Regions with stronger functional connectivity exhibit greater covariance in tau-PET change

**Figure 5:**
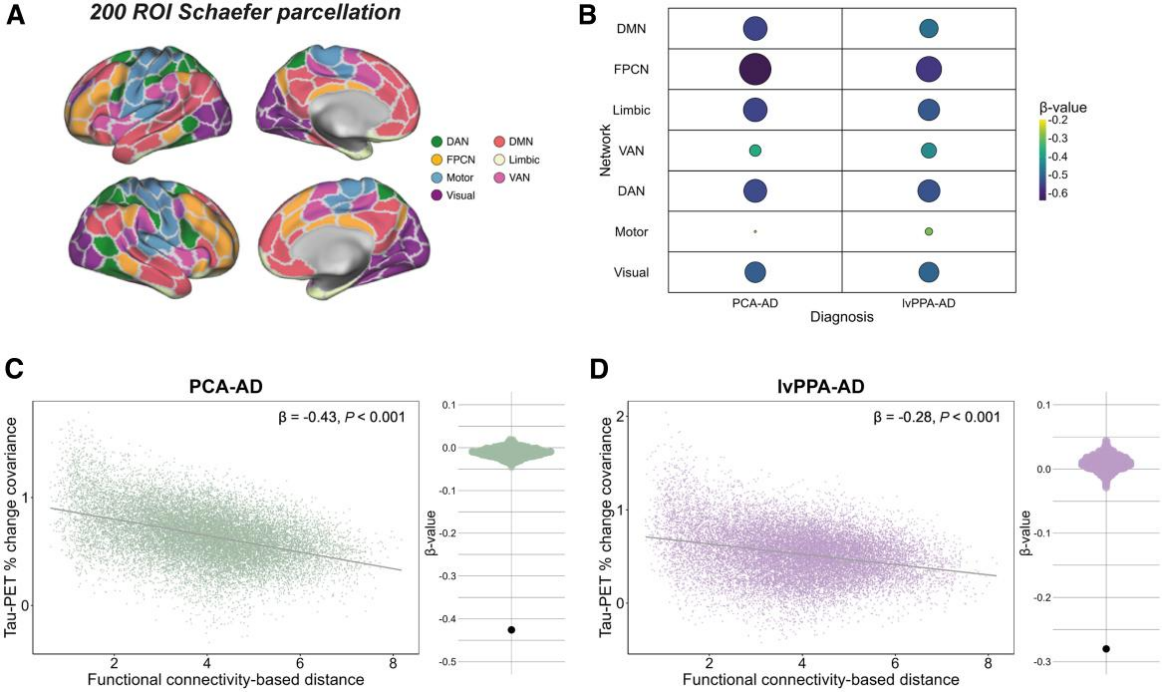
**Association between functional connectivity and covariance in tau-PET percentage change in PCA-AD and lvPPA-AD.** Surface rendering of the 200 ROI brain atlas used for tau-PET and resting-state functional MRI (fMRI) data in ROI-based analyses (**A**). We computed annual tau-PET SUVR change for each individual by fitting 200 linear models (one for each ROI), using follow-up time as the independent variable and tau-PET SUVR as the dependent variable. We then normalized each ROI’s rate of change by the individual’s initial SUVR (at follow-up time = 0) to express it as a relative percentage change per year. Covariance in tau-PET percentage change was determined by calculating AD variant-average Fisher z-transformed partial Pearson correlations between the percentage change rates of all ROI pairs while adjusting for age, sex and site. Using the functional connectivity-based distance matrix described in Fig. 2, we assessed the association between inter-regional functional connectivity-based distance and inter-regional tau-PET percentage change covariance through linear regression, both across the whole brain (**C** and **D**) and in seven individual resting-state fMRI networks separately (**A** and **B**). We re-ran the analysis 1000 times (same procedure as described in Fig. 2) to test the robustness of our findings, as illustrated in the beeswarm panels in **C** and **D**, where the actual *β*-value (furthest data-point) always exceeded the null model *β*-values. AD = Alzheimer’s disease; DAN = dorsal attention network; DMN = default mode network; FPCN = frontoparietal control network; lvPPA = logopenic variant primary progressive aphasia; PCA = posterior cortical atrophy; ROI = region of interest; SUVR = standardized uptake value ratio; VAN = ventral attention network.

Our fourth objective was to test whether higher inter-regional functional connectivity is associated with higher covariance in tau-PET accumulation rates over time. Due to the relatively small sample sizes in the other groups, we only included the PCA-AD and lvPPA-AD groups for our longitudinal analyses. We investigated the association between functional connectivity-based distance across 200 ROIs (as described before) and inter-regional covariance in longitudinal tau-PET change through linear regression. In both PCA-AD and lvPPA-AD, we observed that greater covariance in tau-PET percentage change was associated with shorter functional connectivity-based distance, both across the whole brain (Fig. 5C and D; PCA-AD: *β* = −0.43, *P* < 0.001, lvPPA-AD: *β* = −0.28, *P* < 0.001) and in seven individual resting-state fMRI networks (Fig. 5A and B and Supplementary Fig. 8). When we re-ran the whole-brain analyses 1000 times to test the robustness of our findings, we found that none of the null model-derived *β*-values exceeded the actual *β*-value (Fig. 5C and D). These results indicate that regions with stronger functional connectivity to each other show greater congruence in tau-PET change over time.

### Regions more functionally connected to the tau-PET accumulation epicentre show faster tau-PET change

**Figure 6:**
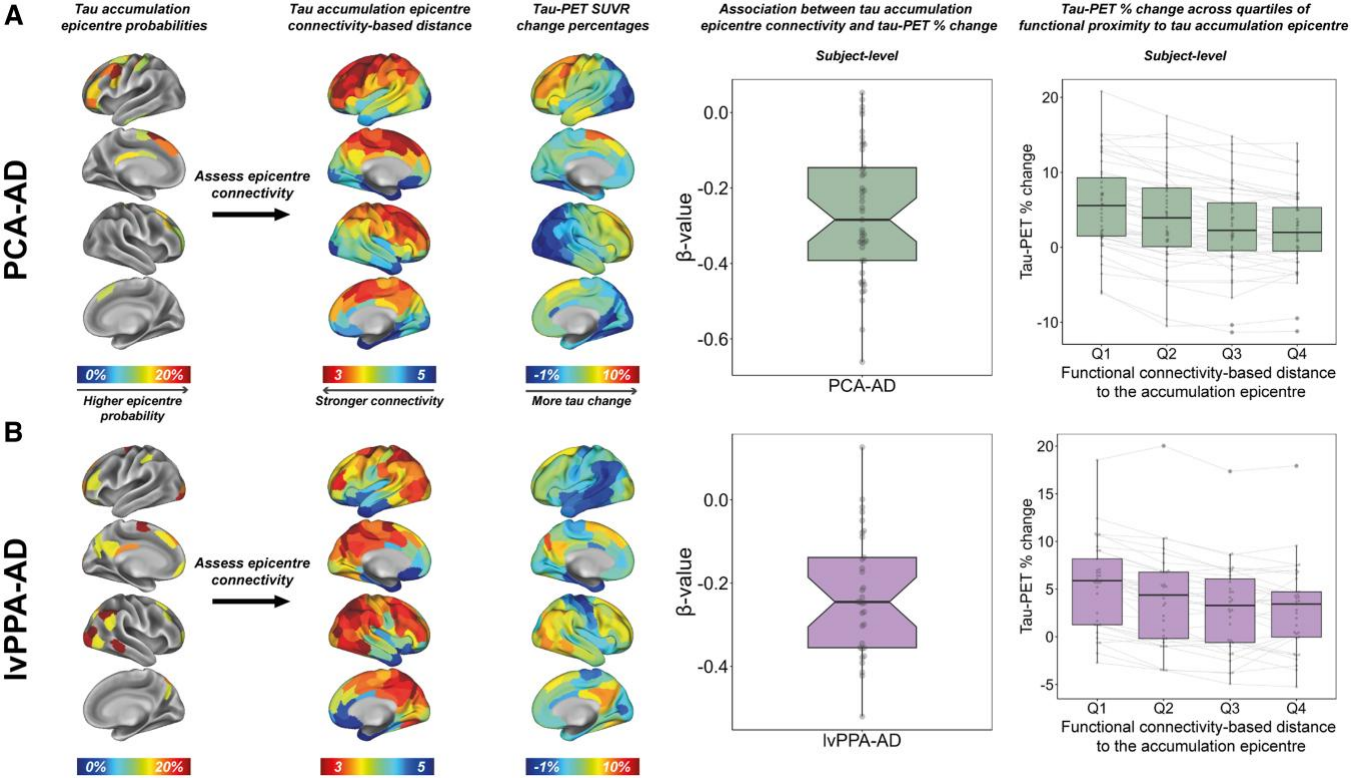
**Association between tau accumulation epicentre connectivity and tau-PET change in PCA-AD and lvPPA-AD.** Tau accumulation epicentres were defined as the top 5% of ROIs (i.e. 10 ROIs in total) with the highest annual percentage change in tau-PET SUVR. Group-average epicentre probabilities indicate the likelihood of a region being part of the epicentre, with only epicentre probabilities ≥10% shown. Tau accumulation epicentre connectivity was determined by taking the functional connectivity-based distance (see Fig. 2 for method specifications) of each non-accumulation-epicentre ROI (*n* = 190) to the accumulation epicentre (*n* = 10). For each individual, linear regression was used to assess the association between functional connectivity-based distance to the tau accumulation epicentre and tau-PET annual percentage change. Subject-level *β*-values are visualized per AD variant in the notched boxplots in **A** and **B**. Additionally, all non-accumulation-epicentre regions were grouped into quartiles based on their functional proximity to the accumulation epicentre (quartile 1 = shortest functional connectivity-based distance, quartile 4 = longest functional connectivity-based distance), and tau-PET percentage change rates across quartiles were compared using paired Wilcoxon signed-rank tests. AD = Alzheimer’s disease; lvPPA = logopenic variant primary progressive aphasia; PCA = posterior cortical atrophy; Q = quartile; ROI = region of interest; SUVR = standardized uptake value ratio.

Our fifth objective was to establish whether functional connectivity of tau-PET accumulation epicentres predicts faster longitudinal increases in tau. We thus aimed to evaluate whether brain regions in closer functional proximity to the tau-PET accumulation epicentre exhibited more tau-PET change than functionally more remote regions. Therefore, we first determined the tau-PET accumulation epicentre for each individual, i.e. the top 5% of regions exhibiting the highest annual tau-PET SUVR percentage change. Then, for PCA-AD and lvPPA-AD separately, we used linear regression to assess the association between subject-level tau accumulation epicentre connectivity and tau-PET percentage change over time. Our results revealed that tau accumulation predominantly occurred anteriorly in PCA-AD (Fig. 6A). Moreover, in lvPPA-AD it was primarily observed in right temporoparietal and occipital regions (Fig. 6B), which likely reflects the close functional connectivity of these regions to the baseline tau-PET epicentres of both variants.^14,51,78^ Moreover, for both PCA-AD and lvPPA-AD, a shorter functional connectivity-based distance to the tau accumulation epicentre was associated with faster tau-PET change, both when tested at the subject level (Fig. 6A and B) and per tracer at the group level (Supplementary Fig. 9). We again divided all brain regions (excluding the tau accumulation epicentre) into quartiles based on their functional proximity to the accumulation epicentre. We then examined whether regions in the lower quartiles (i.e. regions functionally close) showed more tau-PET change than those in the higher quartiles (i.e. regions functionally distant). A gradient of increasing tau-PET change was observed from quartile 4 to quartile 1 for both variants (Fig. 6A and B). Paired Wilcoxon signed-rank tests showed significance (*P* < 0.05) for all quartile comparisons, except for quartile 3 versus quartile 4 in lvPPA-AD. These analyses support our hypothesis that tau propagates across the brain along functional connections, not only in typical AD but also consistently across atypical AD variants with highly heterogeneous tau deposition patterns.

## Discussion

The primary aim of this study was to determine whether connectivity serves as a universal scaffold for predicting tau progression in AD, independent of clinical phenotype or regional predilection of tau deposition. To this end, we conducted a multicentre study combining tau-PET (*n* = 320 cross-sectional, *n* = 78 longitudinal) and post-mortem (*n* = 93) data across 14 sites from various atypical AD variants (i.e. PCA-AD, lvPPA-AD, bvAD and CBS-AD). In line with our primary hypothesis, we found that in all AD variants, brain regions with stronger functional connectivity to each other exhibited greater covariance in concurrent tau-PET deposition and tau-PET change over time. Importantly, this finding was replicated using regionally sampled post-mortem data, wherein we observed that stronger functional connectivity was associated with higher covariance in tau. Furthermore, across all AD variants, brain regions with stronger functional connectivity to the tau-PET epicentre (i.e. the top 5% of regions with the highest tau-PET retention) showed higher tau-PET levels at baseline. Similarly, regions with stronger functional connectivity to the tau-PET accumulation epicentre (i.e. the top 5% of regions with the highest tau accumulation over time) demonstrated faster rates of longitudinal tau-PET accumulation. Taken together, these findings support the hypothesis that tau progresses throughout the brain along functional connections, although spatial progression may also reflect shared vulnerability of connected regions to activity-dependent stressors and proteostasis.^28^ Importantly, connectivity-related progression seems to be consistent across AD phenotypes, establishing functional connectivity as a universal framework for tau progression and highlighting the heightened vulnerability of highly connected brain networks to tau pathology in AD.^79^

Our finding that strongly functionally connected brain regions show correlated tau levels and tau accumulation, and that the functional connectivity of tau epicentres predicts tau progression, aligns with previous studies suggesting that tau pathology propagates through the brain in a prion-like manner, spreading along synaptic connections from cell to cell.^10,12,80,81^ While functional connectivity reflects coordinated activity between brain regions, it is also related to structural connectivity, which can be assessed by methods like diffusion tensor imaging (DTI).^82^ DTI, despite the limitation to accurately capture U-fibres and crossing fibres, reflects anatomical links between areas.^8,82^ Structurally connected regions often show strong functional connectivity, as direct physical pathways facilitate efficient communication.^82^ These structural connections likely serve as routes for the trans-synaptic spread of tau pathology, with both functional and structural networks jointly explaining the observed spatiotemporal patterns of tau accumulation.^15^ Examples of trans-neuronal tau spread have been demonstrated in cellular models of tauopathy, where tau aggregates, or ‘seeds,’ can be released from donor cells, subsequently taken up by recipient cells and then trigger the aggregation of normally soluble tau.^9,22,23,83-88^ This transcellular transfer mechanism is also observed in transgenic or supraphysiological animal models, where tau injections into specific brain regions lead to the emergence of tau pathology in connected areas, reinforcing the concept of network-based propagation.^11,24-26,89-97^ Recent human neuroimaging studies align with these preclinical findings, showing that tau pathology progresses from localized epicentres—proposed to harbour the earliest and highest levels of tau—to connected brain regions.^5,7,13,16-21,27,98^ However, these studies have primarily shown that brain connectivity predicts tau progression in typical amnestic AD, where tau follows the stereotypical Braak staging scheme.^1^ To address this limitation, we included atypical AD variants, each of which display unique tau deposition patterns.^40^ By doing so, our findings provide novel insights into the mechanisms of tau pathology, showing that tau progresses along functional brain connections—also in atypical AD. This supports the universality of network-based tau progression across diverse AD phenotypes and offers a broader framework for understanding tau propagation in complex and less predictable cases of AD.

An unexpected finding was the predominance of tau pathology in the posterior and temporal cortices in the CBS-AD group, contrasting with previous studies that showed significant involvement of the sensorimotor cortex.^40,42^ However, compared with the other AD variants, CBS-AD exhibited greater tau burden in the sensorimotor cortex, suggesting that it was relatively more affected in CBS-AD despite prominent tau accumulation in classical AD regions. This became even more evident when stratifying by lateralization of the clinical symptoms, as subgroup analyses revealed subtle asymmetric tau deposition, with greater tau accumulation in the sensorimotor cortex contralateral to the clinically affected body side. Moreover, since CBS-AD is not limited to motor symptoms, the posterior tau pathology may underlie other clinical features commonly seen in CBS-AD, such as apraxia or visuospatial deficits.^38^

Given that tau is a key driver of neurodegeneration and cognitive decline in AD,^2-4^ our findings have significant implications for personalized medicine and clinical trial design. Understanding the mechanisms and patterns of tau propagation can refine both the timing and application of anti-tau therapies. Predicting which brain regions are most vulnerable to tau spread could enable earlier interventions to halt the cascade of neurodegeneration before critical brain areas are affected. In clinical settings, advanced imaging and computational modelling could be used to identify these at-risk regions, allowing clinicians to anticipate the trajectory of tau spread and strategically time interventions. Administering anti-tau therapies before the pathology compromises key brain areas could help preserve cognitive function and slow disease progression. These insights are particularly relevant for clinical trials, where addressing the heterogeneity across AD variants is a major challenge. Since different AD phenotypes show distinct patterns of tau pathology,^5,16,20,39-43^ our findings support the use of individualized ROIs rather than one-size-fits-all approaches when tau-PET is used as an outcome measure.^99^ Patient-specific ROIs tailored to functional connectivity and tau pathology patterns could improve trial sensitivity, enhance the detection of treatment effects and ultimately increase the likelihood of successful therapeutic outcomes. While tau-PET is not yet widely available in clinical practice, these findings suggest that it may, in the future, play a role in identifying vulnerable brain regions and monitoring disease progression in the clinic.^100,101^ Combined with functional connectivity measures, tau-PET could provide a valuable tool for guiding clinical decision-making and improving patient care.

A major strength of this study is its large sample size with relatively rare AD phenotypes, recruited from 14 sites worldwide, with baseline and longitudinal tau-PET data as well as post-mortem tau assessments available. Notably, the additional inclusion of post-mortem tau assessments—a feature not present in a previous study on functional connectivity and tau spread in atypical AD^16^—represents a novel aspect of our work. There are also several limitations. First, the use of different tau-PET tracers, scanning protocols and approaches for determining Aβ status across sites posed harmonization challenges. Also, Aβ status thresholds were cohort-specific. However, given the high general concordance between amyloid-PET and CSF (∼90%),^102^ we expect limited impact from the use of different methods for determining Aβ status. Second, as expected based on the young age and atypical clinical presentation,^103^ most individuals in the cohort showed signs of saturation in tau-PET retention at baseline, which prevented the ability to directly model longitudinal tau progression from cross-sectional epicentres. Instead, we adopted a tau-PET accumulation epicentre approach, identifying regions with the highest accumulation of tau over time and examining whether tau progresses along the functional connections of these epicentres.^8^ Third, it is challenging to determine the true origin of tau pathology in the brain (i.e. the epicentre) in symptomatic stages of AD. Although our epicentre approach suggests that regions with the highest tau-PET values are variant-specific in atypical AD, it remains possible that tau initially arises in the medial temporal lobe, as seen in typical AD,^17,104^ but spreads to the neocortex much earlier in atypical variants, leading to the observed differences in tau distribution compared with typical AD. Inter-individual differences in brain architecture may promote this; for example, there is evidence that individuals with developmental disorders, where brain connectivity patterns may need to adapt, are at higher risk of atypical AD manifestations.^105-108^ This could be because their brain networks predispose them to faster spread from the medial temporal lobe to other regions. Fourth, in the post-mortem part of the study, tau pathology was assessed in only one hemisphere per individual, which may not fully capture lateralized pathology, especially in syndromes like lvPPA-AD and CBS-AD where asymmetric neuropathologic distributions are well documented.^36,38^ Fifth, the two sites that provided post-mortem tau data employed different methodologies. Specifically, UPENN used a semi-quantitative approach with PHF-1 staining,^62^ while UCSF utilized a quantitative method with thioflavin-S fluorescent microscopy staining.^61^ These methods measure distinct aspects of tau pathology: PHF-1 does not differentiate between tau species and could therefore be hypothesized to be more aligned with tau-PET, while thioflavin-S particularly measures NFTs.^109-111^ Sixth, the spatial resolution of PET imaging is limited,^112^ which precludes directly investigating trans-synaptic tau spreading. This limitation implies that, even though our findings lend support to preclinical observations from animal and cellular studies, we are unable to make strong mechanistic inferences about tau propagation due to scale differences (macro versus micro) between our experimental design and these preclinical models. In addition, our study offers only indirect evidence for the trans-synaptic tau spreading hypothesis, and alternative mechanisms (e.g. shared regional vulnerability between connected brain regions) may give rise to similar connectivity-dependent patterns of tau progression.^113,114^ Seventh, to ensure consistency with our fMRI analyses and effectively capture functional brain networks, we used the cortical Schaefer atlas^58^ for our tau-PET analyses. However, this choice prevented us from examining tau-PET in subcortical regions, which may be particularly relevant for CBS-AD, where there could be some subcortical involvement.^75,115,116^ Additionally, the high granularity of the 200 ROI atlas may increase the risk of partial volume effects, potentially making regional tau estimates less precise.

In conclusion, the current study provides strong evidence that, in AD, tau progression is predictable according to the brain's functional connections, independent of the clinical phenotype and the topography of tau load. Future research is warranted in several key areas, including: (i) advancing and validating functional connectivity-based models to more accurately predict individual levels of tau progression, ideally utilizing subject-level fMRI, because such mechanistic understanding will be crucial for identifying potential novel drug targets aimed at slowing or preventing tau accumulation; (ii) elucidating the role of Aβ burden in shaping tau progression patterns across AD variants. Although direct examination was not possible due to differences in modalities for defining Aβ status and the use of different amyloid-PET tracers, prior work in e.g. PCA and lvPPA suggests that regional Aβ deposition may influence local tau accumulation.^117,118^ Therefore, future studies with harmonized approaches will be critical to clarify this relationship; (iii) investigating interactions between tau and other proteinopathies, such as α-synuclein and TAR DNA-binding protein 43 (TDP-43), as these co-pathologies may influence tau propagation and regional vulnerability^21,119,120^; and (iv) improving the clinical characterization of atypical AD phenotypes,^46^ which could facilitate the recruitment of larger and more well-characterized cohorts and enable more uniform scanning protocols and tau-PET tracers. Collectively, these advancements will refine our understanding of tau dynamics, enhance the translational potential of this research for therapeutic development, inform clinical trial design and eventually aid in improving patient care.

## Supplementary Material

awaf279_Supplementary_Data

## Data Availability

Due to the multicentre design of the study, access to individual participant data from each cohort will have to be made available through the principal investigators of the respective cohorts. Generally, anonymized data can be shared by request from qualified academic investigators for the purpose of replicating procedures and results presented in the article, if data transfer is in agreement with the data protection regulation at the institution and is approved by the local Ethics Review Board.
