# Supplementary material for: Connectivity as a universal predictor of tau progression in atypical Alzheimer’s disease
Source: Brain. 2025 Aug 14;148(11):3893–912. doi: 10.1093/brain/awaf279 (PMC12588720; doi:10.1093/brain/awaf279)
Supplement: awaf279_Supplementary_Data [file awaf279_supplementary_data.pdf]

Supplementary methods

Transformation of tau-PET SUVRs to tau positivity probabilities

We transformed tau-PET standardized uptake value ratio (SUVR) values to a uniform probabilistic scale to harmonize the different tracers present in our multicenter cohort. Following a previously described approach,[1,2] we applied two-component Gaussian mixture modeling to the SUVR values of each of the 200 Schaefer atlas regions of interest (ROIs) separately and independently for each tracer. This yielded a bimodal distribution in each ROI, consisting of a normally distributed Gaussian component presumably reflecting off-target and age-related binding, and a skewed component reflecting on-target binding. For every individual and ROI, we computed the probability that the observed SUVR value belonged to the on-target component. As a result, each ROI in every individual was assigned a tau positivity probability, ranging from 0% to 100%. By transforming SUVRs into tau positivity probabilities, this approach minimizes the influence of e.g., tracer-specific off-target binding patterns or different dynamic ranges, enabling the harmonization and pooling of tau-PET data across the 14 different sites. To ascertain that inter-site variability had not influenced the tau positivity probabilities, we validated our approach by applying Gaussian mixture modeling 14 times, each time omitting a different site. This process resulted in 14 datasets, each containing tau positivity probability values per ROI for each individual. We then calculated an average tau positivity probability value per ROI per individual across these datasets. This average tau positivity probability was compared with the original dataset using Pearson correlations. Finally, we applied Levene's test to the 14 datasets to assess the equality of variances across all leave-one-site-out scenarios.

Identification of tau-PET accumulation epicentres

To determine the tau-PET accumulation epicentre for each individual, we employed the same method as for the tau-PET epicentres. This time, however, at the subject level, we rank-ordered all Schaefer ROIs according to their tau-PET rate of change. Subject-specific tau accumulation epicentres were then defined as the top 5% of ROIs (i.e., 10 ROIs in total) with the highest rates of change, reflecting the regions that showed the fastest tau accumulation over time. We opted for this accumulation epicentre method because individuals in the current sample were typically captured during advanced stages of their pathological disease process, reflected by the presence of extensive tau-PET load already at baseline. By focusing on regions with the most rapid tau accumulation, we aimed to identify areas that best reflect ongoing pathological progression in this advanced stage.

Post-mortem assessment

For the University of Pennsylvania (UPENN) cohort, the left hemisphere was assessed in n = 34 individuals (posterior cortical atrophy due to Alzheimer's disease/PCA-AD n = 7, logopenic variant primary progressive aphasia/lvPPA-AD n = 11, behavioural variant AD/bvAD n = 8, corticobasal syndrome/CBS-AD n = 8), and the right hemisphere in n = 29 (PCA-AD n = 5, lvPPA-AD n = 12, bvAD n = 5, CBS-AD n = 7). For the University of California, San Francisco (UCSF) cohort, the left hemisphere was assessed in n = 16 individuals (PCA-AD n = 2, lvPPA-AD n = 8, bvAD n = 3, CBS-AD n = 3), and the right hemisphere in n = 14 (PCA-AD n = 5, lvPPA-AD n = 1, bvAD n = 7, CBS-AD n = 1).

References

1 Franzmeier N, Dewenter A, Frontzkowski L, et al. Patient-centered connectivity-based prediction of tau pathology spread in Alzheimer's disease. *Sci Adv*. 2020;6:eabd1327.

2 Vogel JW, Iturria-Medina Y, Strandberg OT, et al. Spread of pathological tau proteins through communicating neurons in human Alzheimer's disease. *Nat Commun*. 2020;11:2612.

	Amsterdam	Cambridge	Cologne	Leipzig	Lund	Mayo	MGH	McGill	Munich	UCL	UCSF	UPENN	Washington	Yale	*P*
N	9	25	18	19	18	20	28	79	45	7	34	60	15	11	
**Age, yrs**[1]	63.56 ± 5.32[i]	73.72 ± 6.62[klmn]	65.89 ± 6.34[i]	68.11 ± 10.21	69.10 ± 8.63	65.75 ± 7.31[i]	68.73 ± 7.78	68.74 ± 8.97[kl]	73.76 ± 9.78[acfklmn]	65.00 ± 4.28	63.12 ± 8.38[bhi]	62.73 ± 6.27[bhi]	64.20 ± 6.89[bi]	63.64 ± 8.35[bi]	<0.001
**Female**[2]	5 (55.6)	11 (44.0)	13 (72.2)	8 (42.1)	10 (55.6)	11 (55.0)	15 (53.6)	47 (59.5)	23 (51.1)	4 (57.1)	16 (47.1)	30 (50.0)	10 (66.7)	7 (63.6)	0.842
**Education, yrs**[1]	12.56 ± 2.40[gk]	12.52 ± 3.08[fgkl]	NA	NA	12.50 ± 3.52[fgkl]	16.15 ± 2.96[be]	16.96 ± 2.59[abeh]	14.49 ± 3.76[g]	NA	NA	16.41 ± 3.04[abe]	16.05 ± 2.62[be]	15.07 ± 1.87	15.73 ± 2.37	<0.001
**Diagnosis**[2]															<0.001[o]
PCA-AD	5 (55.6)	0 (0.0)	8 (44.4)	9 (47.4)	6 (33.3)	10 (50.0)	14 (50.0)	19 (24.1)	3 (6.7)	7 (100.0)	21 (61.8)	22 (36.7)	9 (60.0)	6 (54.5)	
lvPPA-AD	1 (11.1)	0 (0.0)	9 (50.0)	5 (26.3)	7 (38.9)	10 (50.0)	11 (39.3)	13 (16.5)	2 (4.4)	0 (0.0)	13 (38.2)	21 (35.0)	6 (40.0)	5 (45.5)	
bvAD	2 (22.2)	0 (0.0)	1 (5.6)	0 (0.0)	2 (11.1)	0 (0.0)	1 (3.6)	18 (22.8)	0 (0.0)	0 (0.0)	0 (0.0)	11 (18.3)	0 (0.0)	0 (0.0)	
CBS-AD	1 (11.1)	9 (36.0)	0 (0.0)	5 (26.3)	3 (16.7)	0 (0.0)	0 (0.0)	0 (0.0)	19 (42.2)	0 (0.0)	0 (0.0)	6 (10.0)	0 (0.0)	0 (0.0)	
Typical AD	0 (0.0)	16 (64.0)	0 (0.0)	0 (0.0)	0 (0.0)	0 (0.0)	2 (7.1)	29 (36.7)	21 (46.7)	0 (0.0)	0 (0.0)	0 (0.0)	0 (0.0)	0 (0.0)	
***APOE*ε4 carrier**[2]	4 (44.4)	8 (66.7)	NA	2 (50.0)	12 (66.7)	9 (45.0)	3 (42.9)	46 (59.7)	NA	NA	17 (53.1)	21 (41.2)	NA	5 (62.5)	0.557
**MMSE**[3]	20.00 ± 3.87	25.12 ± 4.98[hk]	23.35 ± 4.14	19.58 ± 8.90	23.00 ± 4.37	NA	21.96 ± 5.32	21.34 ± 5.78[b]	26.50 ± 2.20	22.00 ± 4.04	20.74 ± 4.94[b]	22.80 ± 5.03	20.25 ± 6.07	18.10 ± 8.08	0.002

Supplementary Table 1: Tau-PET cohort – demographic and clinical information across participating sites. Values are mean ± standard deviation for continuous variables and *n* (%) for categorical variables. Differences between groups were assessed using ANOVA,[1] Chi-squared tests of independence,[2] and Kruskal-Wallis test.[3] In case of cell counts <5, Monte Carlo simulations with 20,000 replications (B = 20,000) were employed to estimate the P-values for the Chi-squared tests. If a statistically significant main effect was observed, Tukey's Honestly Significant Difference (HSD) test was used as post hoc test following ANOVA, Fisher's Exact tests following Chi-square tests, and Dunn's test following Kruskal-Wallis test. Fisher's Exact tests and Dunn's test were adjusted for multiple comparisons using the Bonferroni correction. When data was missing for a category (education n = 89, *APOE*ε4 status n = 150, MMSE n = 79), individuals were excluded from that specific analysis. [a]Significantly different from Amsterdam. [b]Significantly different from Cambridge. [c]Significantly different from Cologne. [d]Significantly different from Leipzig. [e]Significantly different from Lund. [f]Significantly different from Mayo. [g]Significantly different from MGH. [h]Significantly different from McGill. [i]Significantly different from Munich. [j]Significantly different from UCL. [k]Significantly different from UCSF. [l]Significantly different from UPENN. [m]Significantly different from Washington. [n]Significantly different from Yale. [o]Significant differences: Cambridge vs UCSF, Cambridge vs UPENN, Munich vs UPENN, Munich vs UCSF, Cambridge vs Mayo, Cambridge vs MGH, McGill vs Munich, Mayo vs Munich, Cambridge vs Cologne, MGH vs Munich, Cologne vs Munich, Cambridge vs Washington, Cambridge vs McGill, Munich vs Washington, Cambridge vs Yale, McGill vs UPENN, McGill vs UCSF, Cambridge vs Lund, Cambridge vs Leipzig, Munich vs Yale, Leipzig vs McGill, Lund vs Munich, Cambridge vs UCL, Amsterdam vs Cambridge, Leipzig vs Munich, Munich vs UCL, Mayo vs McGill, Amsterdam vs Munich, Lund vs McGill, MGH vs McGill, McGill vs Washington, Cologne vs McGill. AD = Alzheimer's disease; *APOE* = apolipoprotein E; bvAD = behavioural variant Alzheimer's disease; CBS = corticobasal syndrome; lvPPA = logopenic variant primary progressive aphasia; MGH = Massachusetts general hospital; MMSE = mini-mental state examination; NA = not applicable; PCA = posterior cortical atrophy; PET = positron emission tomography; UCL = university college London; UCSF = university of California, San Francisco; UPENN = university of Pennsylvania; yrs = years.

**AD variant**	***F*-value**	***P*-value**
PCA-AD	0.328	0.991
lvPPA-AD	0.410	0.972
bvAD	1.191	0.275
CBS-AD	1.083	0.368
Typical AD	0.836	0.630

Supplementary Table 2: Levene's test results assessing variance stability in tau positivity probabilities across leave-one-site-out scenarios. To assess whether inter-site variability introduced systematic differences in tau positivity probabilities, we validated our approach by applying Gaussian mixture modeling 14 times, each time omitting a different site. This process generated 14 datasets, each containing tau positivity probability values per region for every individual. We applied Levene's test to assess the equality of variances across all these leave-one-site-out scenarios. AD = Alzheimer's disease; bvAD = behavioural variant Alzheimer's disease; CBS = corticobasal syndrome; lvPPA = logopenic variant primary progressive aphasia; PCA = posterior cortical atrophy.

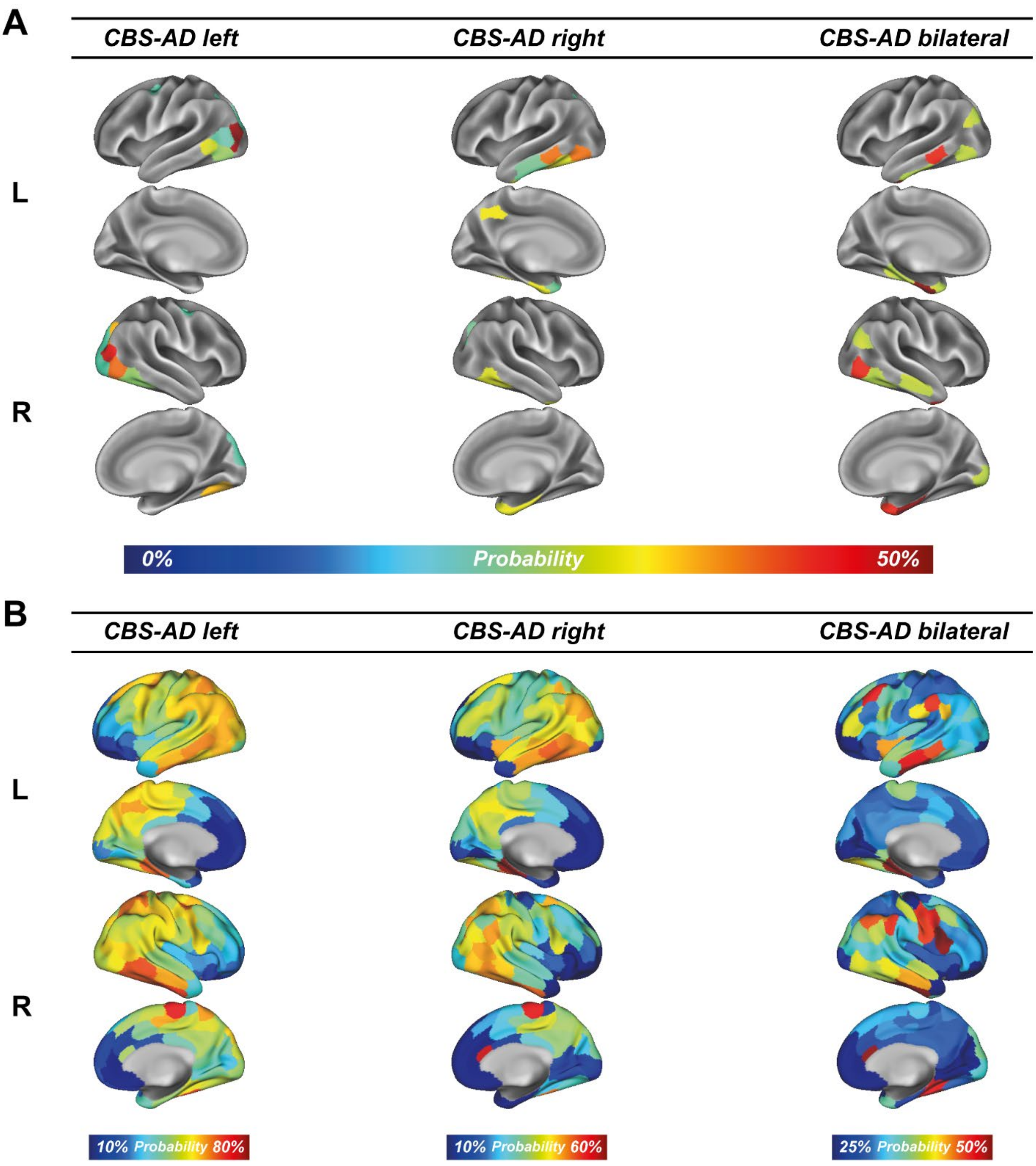

Supplementary Figure 1: Tau-PET epicentres and positivity across CBS-AD variants. Tau epicentres (**A**) were defined at the subject level as the 5% regions with the highest tau-PET SUVRs at baseline. CBS-AD (predominant clinical symptoms left/right/bilateral) group-average epicentre probabilities indicate the likelihood of a region being part of the epicentre, with only epicentre probabilities ≥20% shown. Group-average tau-PET positivity probability mapping across CBS-AD variants are shown in (**B**). AD = Alzheimer's disease; CBS = corticobasal syndrome; L = left; PET = positron emission tomography; R = right; SUVR = standardized uptake value ratio.

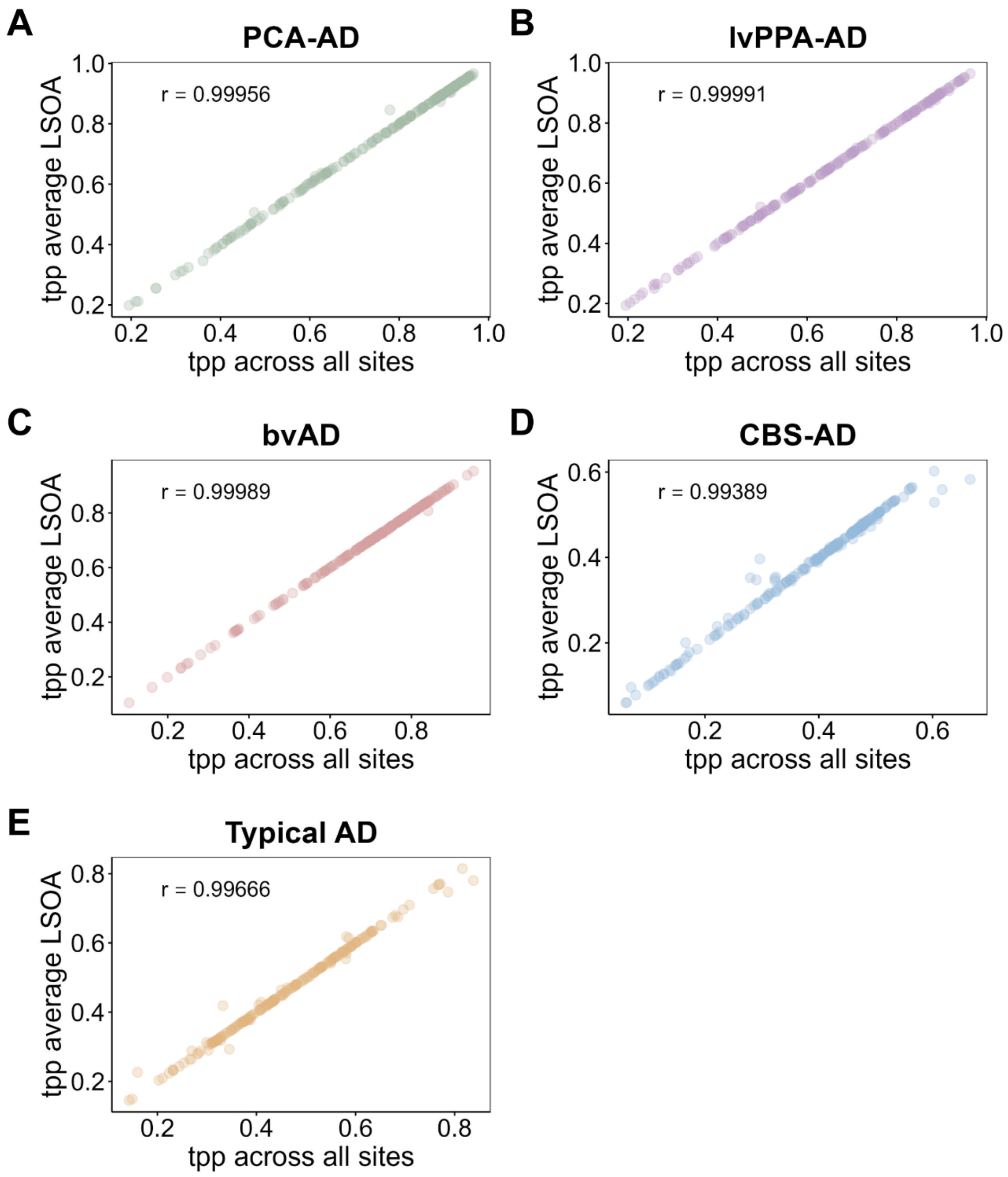

Supplementary Figure 2: Correlation between tau positivity probabilities from standard and leave-one-site-out approaches. Using the 14 leave-one-site-out datasets described in **Suppl. Table 2**, we calculated the average tau positivity probability value per region per individual across all these datasets. This final result was compared with the original dataset using Pearson correlations. AD = Alzheimer's disease; bvAD = behavioural variant Alzheimer's disease; CBS = corticobasal syndrome; LSOA = leave-one-site-out approach; lvPPA = logopenic variant primary progressive aphasia; PCA = posterior cortical atrophy; tpp = tau positivity probability.

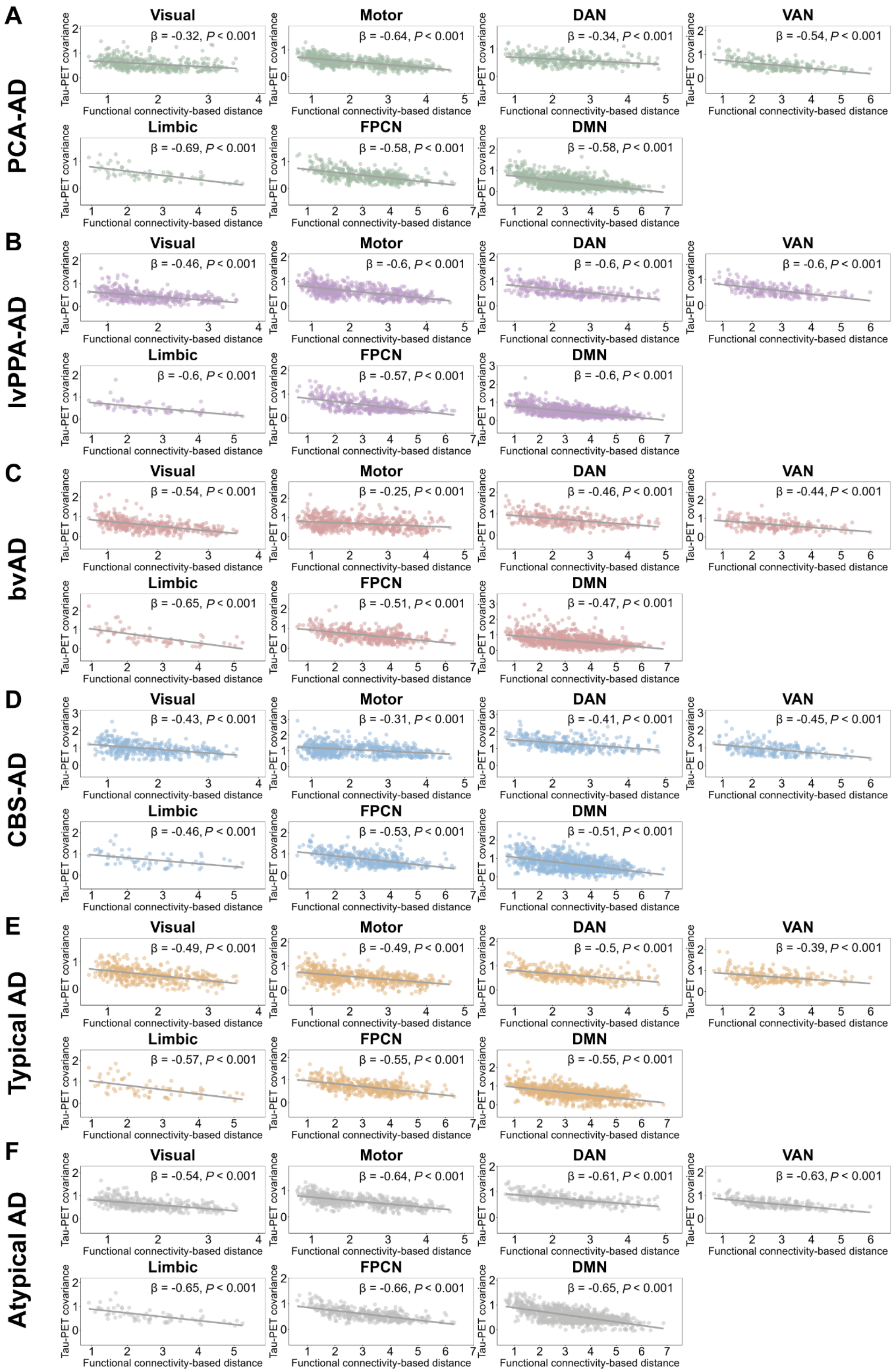
A
PCA-AD
Visual β = -0.32, P < 0.001
Motor β = -0.64, P < 0.001
DAN β = -0.34, P < 0.001
VAN β = -0.54, P < 0.001
Limbic β = -0.69, P < 0.001
FPCN β = -0.58, P < 0.001
DMN β = -0.58, P < 0.001
B
lvPPA-AD
Visual β = -0.46, P < 0.001
Motor β = -0.6, P < 0.001
DAN β = -0.6, P < 0.001
VAN β = -0.6, P < 0.001
Limbic β = -0.6, P < 0.001
FPCN β = -0.57, P < 0.001
DMN β = -0.6, P < 0.001
C
bvAD
Visual β = -0.54, P < 0.001
Motor β = -0.25, P < 0.001
DAN β = -0.46, P < 0.001
VAN β = -0.44, P < 0.001
Limbic β = -0.65, P < 0.001
FPCN β = -0.51, P < 0.001
DMN β = -0.47, P < 0.001
D
CBS-AD
Visual β = -0.43, P < 0.001
Motor β = -0.31, P < 0.001
DAN β = -0.41, P < 0.001
VAN β = -0.45, P < 0.001
Limbic β = -0.46, P < 0.001
FPCN β = -0.53, P < 0.001
DMN β = -0.51, P < 0.001
E
Typical AD
Visual β = -0.49, P < 0.001
Motor β = -0.49, P < 0.001
DAN β = -0.5, P < 0.001
VAN β = -0.39, P < 0.001
Limbic β = -0.57, P < 0.001
FPCN β = -0.55, P < 0.001
DMN β = -0.55, P < 0.001
F
Atypical AD
Visual β = -0.54, P < 0.001
Motor β = -0.64, P < 0.001
DAN β = -0.61, P < 0.001
VAN β = -0.63, P < 0.001
Limbic β = -0.65, P < 0.001
FPCN β = -0.66, P < 0.001
DMN β = -0.65, P < 0.001
Tau-PET covariance
Functional connectivity-based distance

Supplementary Figure 3: Association between functional connectivity and covariance in tau-PET within seven resting-state functional MRI networks across AD variants. Tau-PET covariance was defined as AD variant-average Fisher z-transformed partial Pearson correlations between tau positivity probabilities of all possible ROI pairs, while adjusting for age, sex, and site. Using the functional connectivity-based distance matrix described in **Fig. 2**, we assessed the association between inter-regional functional connectivity-based distance and inter-regional tau-PET covariance through linear regression within seven resting-state functional MRI networks for all AD variants (**A-F**). AD = Alzheimer's disease; bvAD = behavioural variant Alzheimer's disease; CBS = corticobasal syndrome; DAN = dorsal attention network; DMN = default mode network; FPCN = frontoparietal control network; lvPPA = logopenic variant primary progressive aphasia; PCA = posterior cortical atrophy; PET = positron emission tomography; ROI = region of interest; VAN = ventral attention network.

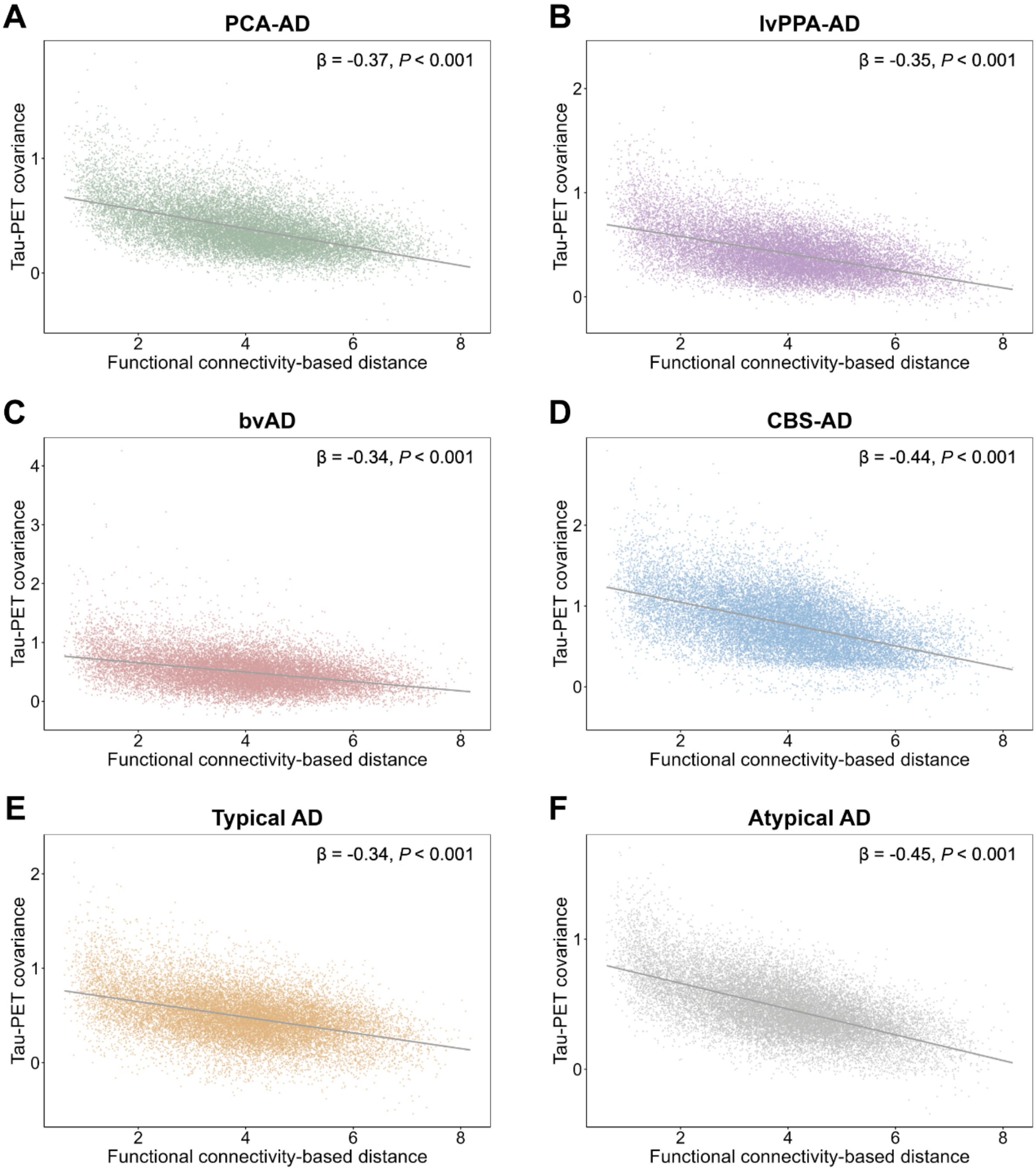

Supplementary Figure 4: Association between functional connectivity and covariance in tau-PET in variants of AD. Tau-PET covariance was defined as AD variant-average Fisher z-transformed partial Pearson correlations between tau positivity probabilities of all possible ROI pairs, while adjusting for age, sex, site, and Euclidean distance. Using the functional connectivity-based distance matrix described in **Fig. 2**, we assessed the association between inter-regional functional connectivity-based distance and inter-regional tau-PET covariance through linear regression for all AD variants (**A-F**). AD = Alzheimer's disease; bvAD = behavioural variant Alzheimer's disease; CBS = corticobasal syndrome; lvPPA = logopenic variant primary progressive aphasia; PCA = posterior cortical atrophy; PET = positron emission tomography; ROI = region of interest.

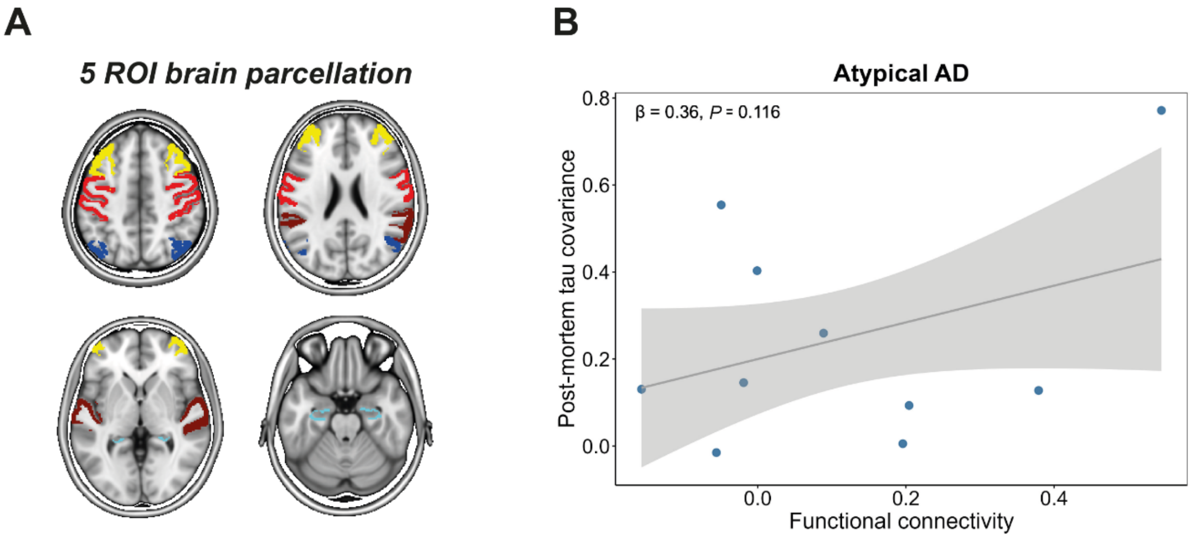

Supplementary Figure 5: Association between functional connectivity and covariance in post-mortem tau pathology in atypical AD. Using established cortical and subcortical brain atlases (i.e., AAL and CoBrA), we created a bilateral MRI brain atlas for the regions where tau pathology rating was available (n = 5, see **A**). Functional connectivity was defined as Fisher z-transformed Pearson correlations between functional MRI time series (reflective of fluctuations in the BOLD signal) of all ROI pairs in 42 CN Aβ-negative individuals from ADNI. Tau covariance was defined as Fisher z-transformed partial Pearson correlations between quantitative tau pathology ratings of all ROI pairs, while adjusting for age and sex. We pooled the data from all AD variants to increase statistical power. The association between functional connectivity and tau pathology covariance was assessed using linear regression (**B**). AAL = automated anatomical labelling; Aβ = amyloid-β; AD = Alzheimer's disease; ADNI = Alzheimer's disease neuroimaging initiative; BOLD = blood oxygen level-dependent; CN = cognitively normal; CoBrA = computational brain anatomy laboratory; ROI = region of interest.

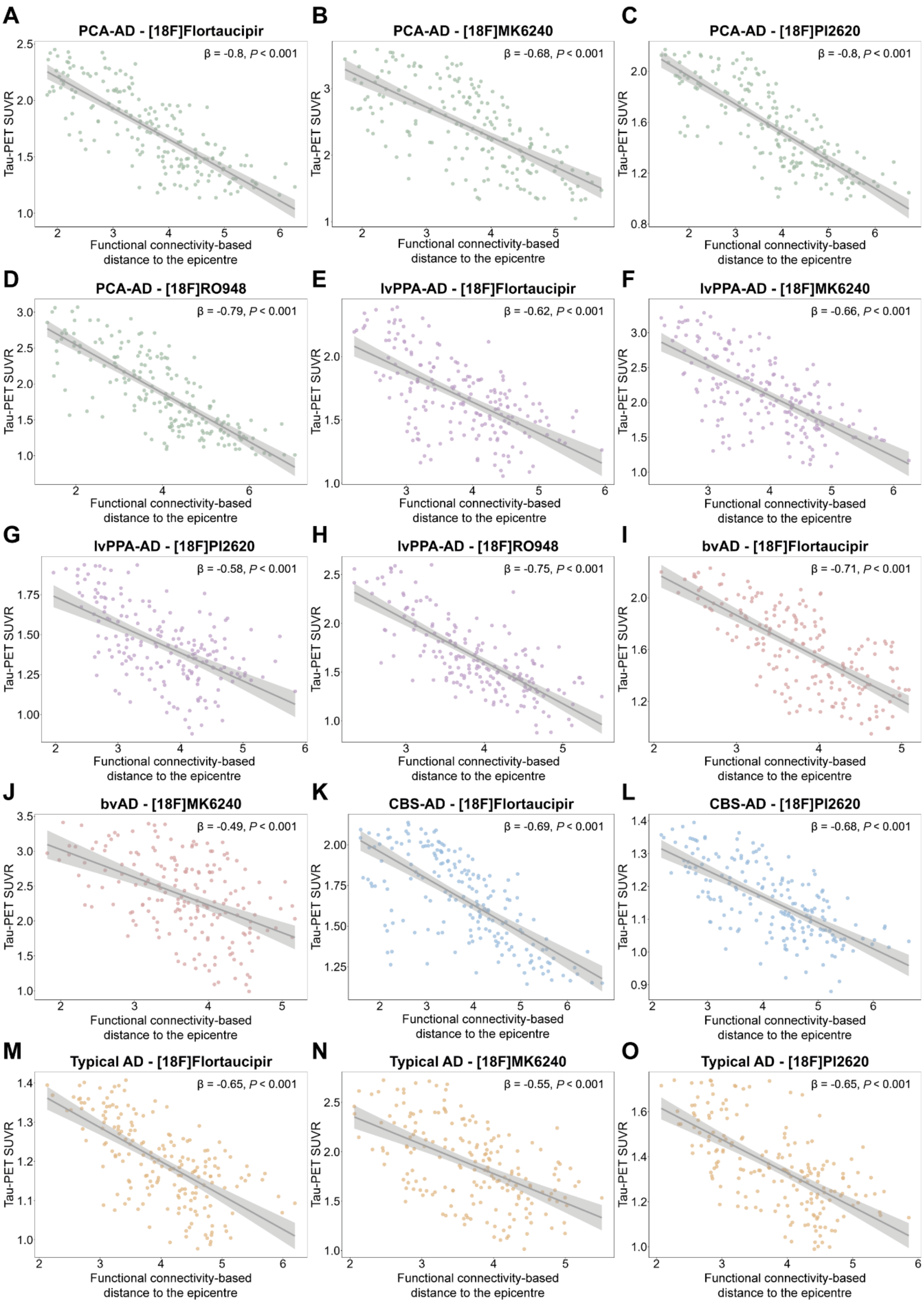
A
PCA-AD - [18F]Flortaucipir
β = -0.8, P < 0.001
B
PCA-AD - [18F]MK6240
β = -0.68, P < 0.001
C
PCA-AD - [18F]PI2620
β = -0.8, P < 0.001
D
PCA-AD - [18F]RO948
β = -0.79, P < 0.001
E
lvPPA-AD - [18F]Flortaucipir
β = -0.62, P < 0.001
F
lvPPA-AD - [18F]MK6240
β = -0.66, P < 0.001
G
lvPPA-AD - [18F]PI2620
β = -0.58, P < 0.001
H
lvPPA-AD - [18F]RO948
β = -0.75, P < 0.001
I
bvAD - [18F]Flortaucipir
β = -0.71, P < 0.001
J
bvAD - [18F]MK6240
β = -0.49, P < 0.001
K
CBS-AD - [18F]Flortaucipir
β = -0.69, P < 0.001
L
CBS-AD - [18F]PI2620
β = -0.68, P < 0.001
M
Typical AD - [18F]Flortaucipir
β = -0.65, P < 0.001
N
Typical AD - [18F]MK6240
β = -0.55, P < 0.001
O
Typical AD - [18F]PI2620
β = -0.65, P < 0.001
Tau-PET SUVR
Functional connectivity-based distance to the epicentre

Supplementary Figure 6: Association between tau epicentre connectivity and group-level tau-PET SUVR across AD variants and tau-PET tracers. Tau epicentre connectivity was determined by taking the functional connectivity-based distance (see **Fig. 2** for method specifications) of each non-epicentre ROI (n = 190) to the epicentre (n = 10). For each AD variant separately, linear regression was used to assess the association between functional connectivity-based distance to the tau epicentre and tau-PET SUVR (per tracer and at the group level). AD = Alzheimer's disease; bvAD = behavioural variant Alzheimer's disease; CBS = corticobasal syndrome; lvPPA = logopenic variant primary progressive aphasia; PCA = posterior cortical atrophy; PET = positron emission tomography; ROI = region of interest; SUVR = standardized uptake value ratio.

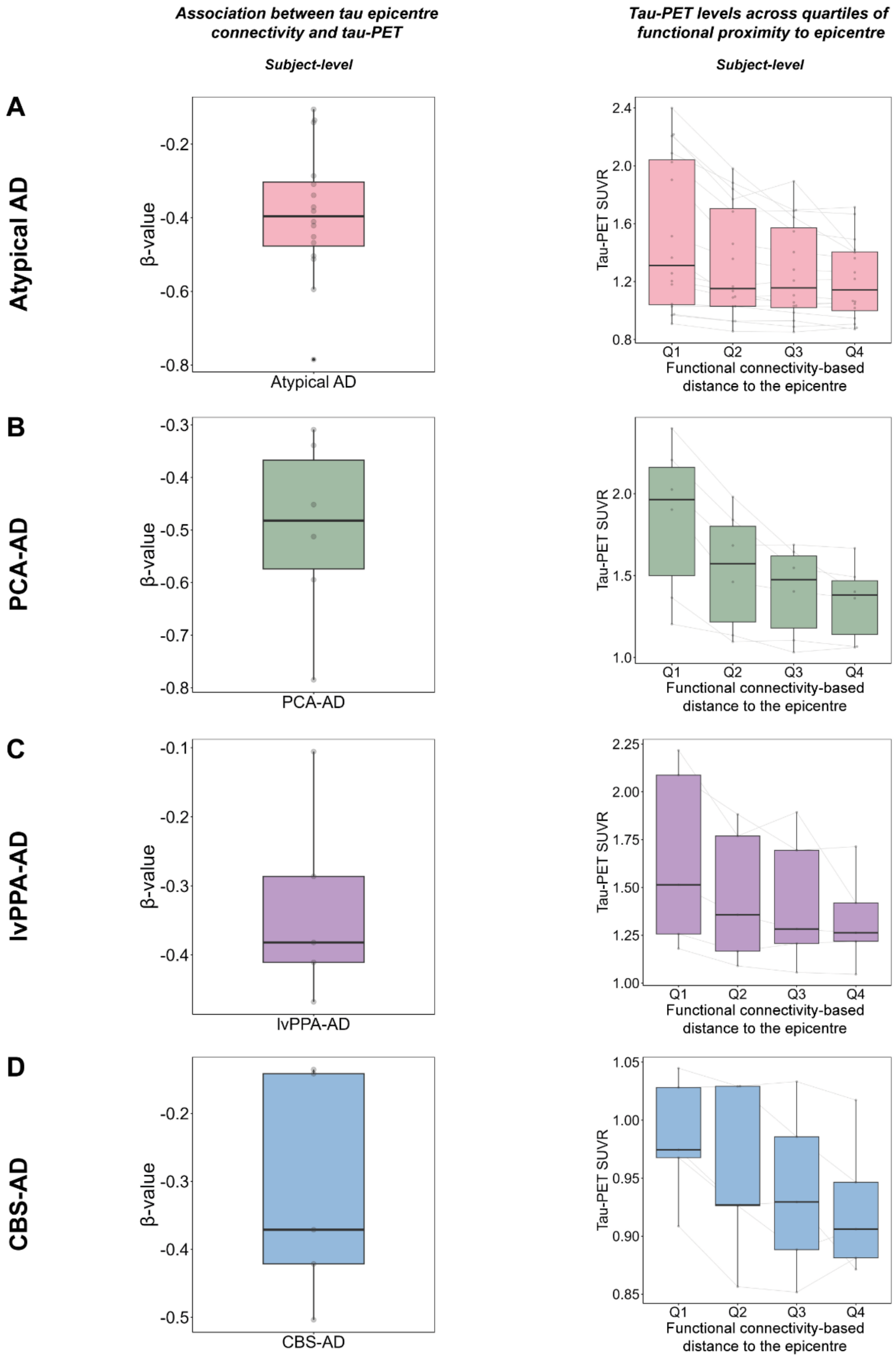
Association between tau epicentre connectivity and tau-PET
Subject-level
Tau-PET levels across quartiles of functional proximity to epicentre
Subject-level
A
Atypical AD
β-value
-0.2
-0.4
-0.6
-0.8
Atypical AD
Tau-PET SUVR
2.4
2.0
1.6
1.2
0.8
Q1
Q2
Q3
Q4
Functional connectivity-based distance to the epicentre
B
PCA-AD
β-value
-0.3
-0.4
-0.5
-0.6
-0.7
-0.8
PCA-AD
Tau-PET SUVR
2.0
1.5
1.0
Q1
Q2
Q3
Q4
Functional connectivity-based distance to the epicentre
C
lvPPA-AD
β-value
-0.1
-0.2
-0.3
-0.4
lvPPA-AD
Tau-PET SUVR
2.25
2.00
1.75
1.50
1.25
1.00
Q1
Q2
Q3
Q4
Functional connectivity-based distance to the epicentre
D
CBS-AD
β-value
-0.2
-0.3
-0.4
-0.5
CBS-AD
Tau-PET SUVR
1.05
1.00
0.95
0.90
0.85
Q1
Q2
Q3
Q4
Functional connectivity-based distance to the epicentre

Supplementary Figure 7: Association between tau epicentre connectivity and tau-PET across AD variants in a subset of individuals from Leipzig with subject-level functional MRI. Tau epicentre connectivity was determined by taking the individualized functional connectivity-based distance of each non-epicentre ROI (n = 190) to the epicentre (n = 10). For each individual, linear regression was used to assess the association between functional connectivity-based distance to the tau epicentre and tau-PET SUVR. Subject-level β-values are visualized per AD variant in the boxplots in **A-D**. Additionally, all non-epicentre regions were grouped into quartiles based on their functional proximity to the epicentre (quartile 1 = close, quartile 4 = distant), and tau-PET SUVRs across quartiles were compared using paired Wilcoxon signed-rank tests. AD = Alzheimer's disease; CBS = corticobasal syndrome; lvPPA = logopenic variant primary progressive aphasia; PCA = posterior cortical atrophy; PET = positron emission tomography; Q = quartile; ROI = region of interest; SUVR = standardized uptake value ratio.

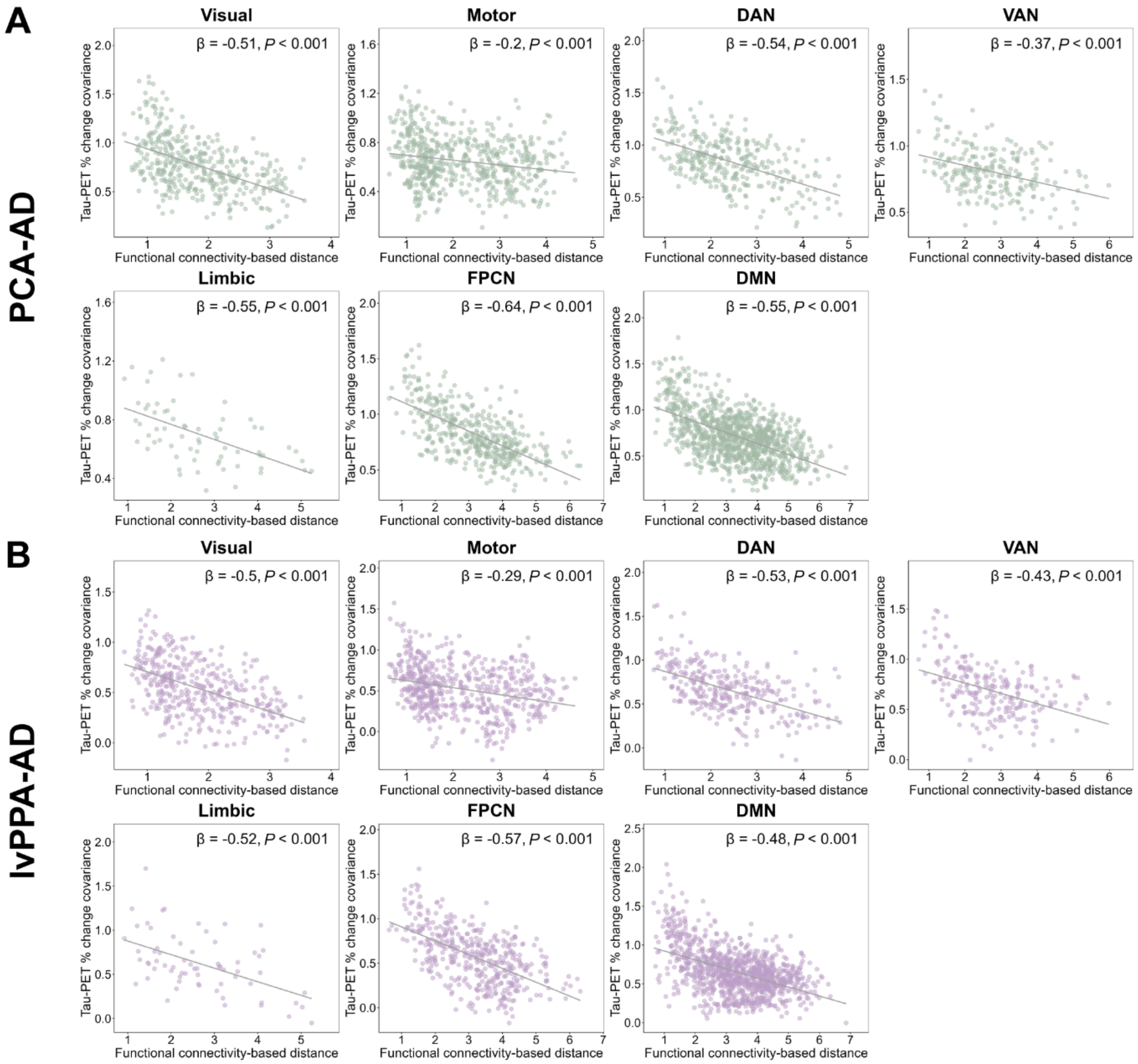

Supplementary Figure 8: Association between functional connectivity and covariance in tau-PET change within seven resting-state functional MRI networks in PCA-AD and lvPPA-AD. Covariance in tau-PET percentage change (see **Fig. 5** for a description of how tau-PET change was computed) was determined by calculating AD variant-average Fisher z-transformed partial Pearson correlations between the percentage change rates of all ROI pairs, while adjusting for age, sex, and site. Using the functional connectivity-based distance matrix described in **Fig. 2**, we assessed the association between inter-regional functional connectivity-based distance and inter-regional tau-PET percentage change covariance through linear regression within seven resting-state functional MRI networks for PCA-AD and lvPPA-AD (**A-B**). AD = Alzheimer's disease; DAN = dorsal attention network; DMN = default mode network; FPCN = frontoparietal control network; lvPPA = logopenic variant primary progressive aphasia; PCA = posterior cortical atrophy; PET = positron emission tomography; SUVR = standardized uptake value ratio; VAN = ventral attention network.

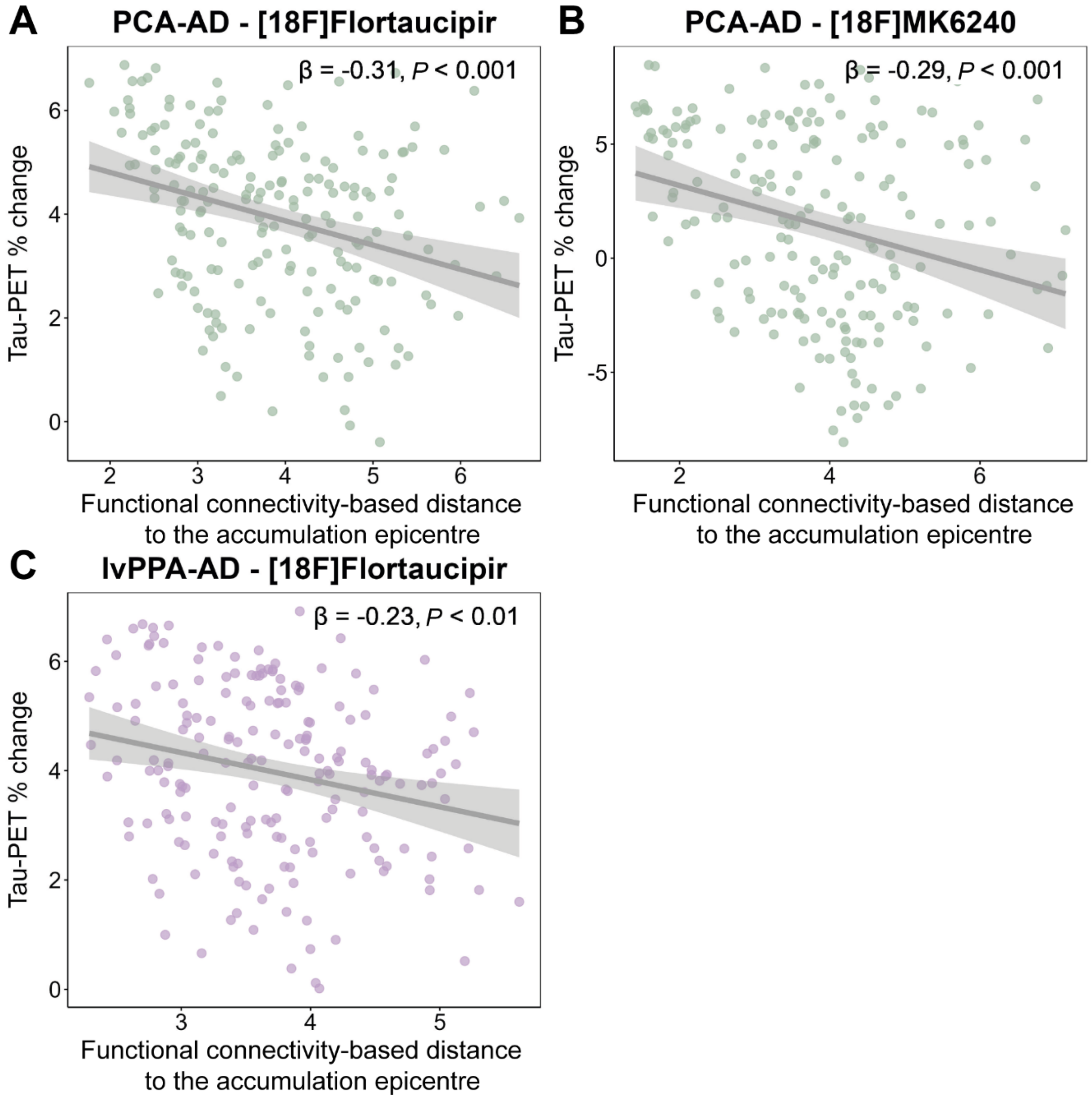

Supplementary Figure 9: Association between tau accumulation epicentre connectivity and group-level tau-PET change in PCA-AD and lvPPA-AD. Tau accumulation epicentre connectivity was determined by taking the functional connectivity-based distance (see **Fig. 2** for method specifications) of each non-accumulation-epicentre ROI (n = 190) to the accumulation epicentre (n = 10). For both PCA-AD and lvPPA-AD, linear regression was used to assess the association between functional connectivity-based distance to the tau accumulation epicentre and tau-PET annual percentage change (computed as described in **Fig. 5**) per tracer at the group level. AD = Alzheimer's disease; lvPPA = logopenic variant primary progressive aphasia; PCA = posterior cortical atrophy; PET = positron emission tomography; ROI = region of interest.
